# A Delocalized Mixed-Valence Dinuclear Ytterbium Complex That Displays Intervalence Charge Transfer

**DOI:** 10.1021/jacs.4c12188

**Published:** 2024-10-14

**Authors:** Tom J. N. Obey, Mukesh K. Singh, Angelos B. Canaj, Gary S. Nichol, Euan K. Brechin, Jason B. Love

**Affiliations:** EaStCHEM School of Chemistry, University of Edinburgh, EH9 3FJ, Edinburgh, United Kingdom

## Abstract

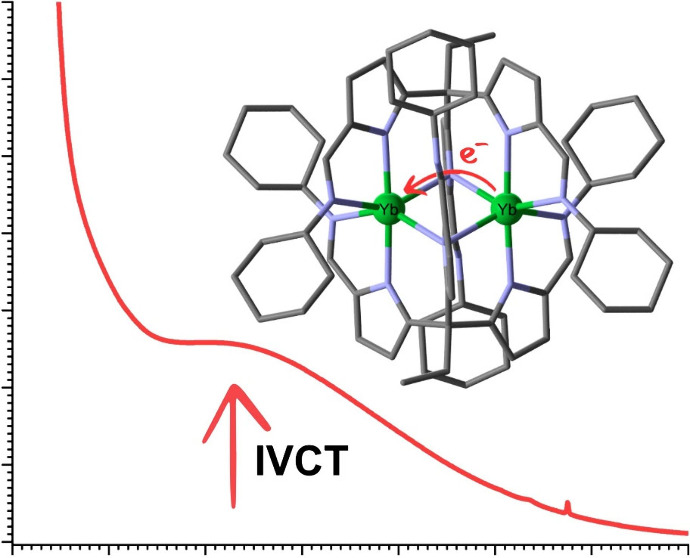

The analysis of intervalence charge transfer (IVCT) in mixed-valence compounds can help understand electron transfer processes that are important in diverse applications such as molecular electronics and artificial photosynthesis. While mixed-valence complexes of the lanthanides are more difficult to access than their transition metal analogues, they have shown IVCT phenomena derived from Robin–Day Class II localized valency or even electronic transitions due to *d*–*d* metal–metal bonding. In contrast, we report here the synthesis, characterization, and computational analysis of a rare, Robin-Day Class III, singly reduced dinuclear Yb complex, which is best viewed as having delocalized oxidation states. In this case, no metal–metal bonding occurs and, for the first time, IVCT in a Robin–Day Class III complex resulting from *f*–*f* transitions is observed.

Mixed-valence complexes comprise two or more metal centers in differing oxidation states. The Robin–Day classification divides these compounds into three classes based on the strength of the electronic interactions between the metals in their different oxidation states.^1^ In Class I, valences are localized on distinct metal sites and interconversion does not occur. Class II compounds display localization of valences but have a low thermal energy barrier to the transfer of the charge between the metal centers. In contrast, Class III compounds exhibit complete delocalization of the charge with the two metal sites indistinguishable. Significantly, Class II and III compounds exhibit intervalence charge transfer (IVCT) absorptions that arise from the transfer of charge from one metal to another.^2^ Analysis of IVCT absorptions allows the derivation of parameters of the electron-transfer process between mixed-valence redox sites, information that is important and applicable to the study of, for example, molecular electronics and artificial photosynthesis.^3^

Mixed-valence complexes of the lanthanides are unsurprisingly rare due to the difficulty in accessing oxidation states other than the common 3+ state. However, there are several reports of molecular mixed-valence complexes of samarium, europium and ytterbium as a result of the accessible Ln^3+^/Ln^2+^ redox potentials for these lanthanides.^4,5^ These cases are generally classified as Class I complexes that exhibit no IVCT or Class II complexes that display differences in the coordination modes of the valence-localized Ln^3+^ and Ln^2+^ sites.

Recently, however, mixed-valence homomultimetallic lanthanide and actinide complexes have been reported that exhibit metal–metal bonding, resulting in properties different to the exclusively 4*f* mixed-valence complexes described above.^6−8^ While metal–metal bonding is not considered in the Robin–Day classification, these unusual compounds are commonly classified as Class III in which *d*-electrons are shared by the metals. In these cases, IVCT absorptions are hard to assign as it is difficult to distinguish between a genuine 4*f* to 4*f* IVCT and a delocalized 5*d* σ-to-σ* transition.^9^ In contrast to the extensively explored transition-metal mixed-valence complexes exhibiting IVCT phenomena, lanthanide mixed-valence complexes displaying IVCT have been less investigated, despite their potential for providing insights into the electronic structure and interactions of these unusual species.^10^

Herein, we report a combined experimental and computational study on a homodinuclear ytterbium(III) complex of a polydentate iminopyrrolide ligand and its single-electron reduction to a Class III mixed-valence compound of Yb^2.5+^ oxidation states that exhibits IVCT. To the best of our knowledge, this is the first Class III mixed-4*f*-valence molecular lanthanide complex that exhibits IVCT in the absence of a metal–metal bond.

The anaerobic reaction between the tripodal ligand, EtC{2,5-C_4_H_2_NHCH=NCy)_3_ (H_3_L) and Yb{N(SiMe_2_)_3_}_3_ in toluene gives the homodinuclear complex {Yb(py)(L)}_2_**1** in 65% crystalline yield (see the Electronic Supporting Information (ESI) and Scheme 1). The X-ray crystal structure of **1** (Figures SI 1 and SI 2, as well as Table SI 1) shows a centrosymmetric dinuclear structure in which two iminopyrrolide ligand arms coordinate to one metal center, with the third extending to the second metal. One pyridine molecule coordinates to each ytterbium center, resulting in a seven-coordinate metal geometry. The two metals are not bridged by any shared atoms and, consequently, the Yb**···**Yb separation is large at 5.6214(5) Å.

**Scheme 1:**
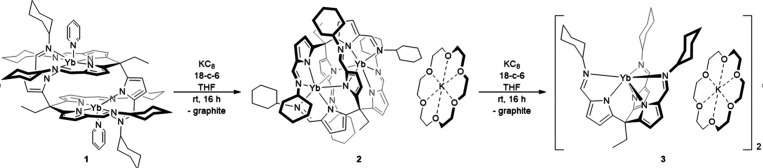
One-Electron Reduction of the Homo-Dinuclear Ytterbium Complex **1** to the Mixed Valence Dinuclear Complex **2**, along with the Further One-Electron Reduction to a Mononuclear Ytterbium(II) Complex, **3**

The addition of one equivalent of potassium graphite and 18-crown-6 to a solution of **1** in THF produces a dark brown solution for which the ^1^H NMR spectrum is silent and from which single crystals of the singly reduced complex [K(18-c-6)][Yb(L)]_2_**2** were isolated in 35% yield (Scheme 1). The X-ray structure of **2** shows a centrosymmetric monoanionic dinuclear Yb complex and a single potassium counterion that is coordinated by 18-crown-6 (see Figure 1; details are given in Tables SI 1 and SI 2). It is evident that single-electron reduction of dinuclear **1** causes a significant rearrangement of the ligand, with one arm on each ligand coordinating to each metal and the third bridging the two metals through the pyrrolide nitrogen atom (N5/N5′), with each metal adopting a pseudo-octahedral geometry. The imine-nitrogen associated with the bridging pyrrolide groups (N6/N6′) is no longer coordinated to a metal.

**Figure 1:**
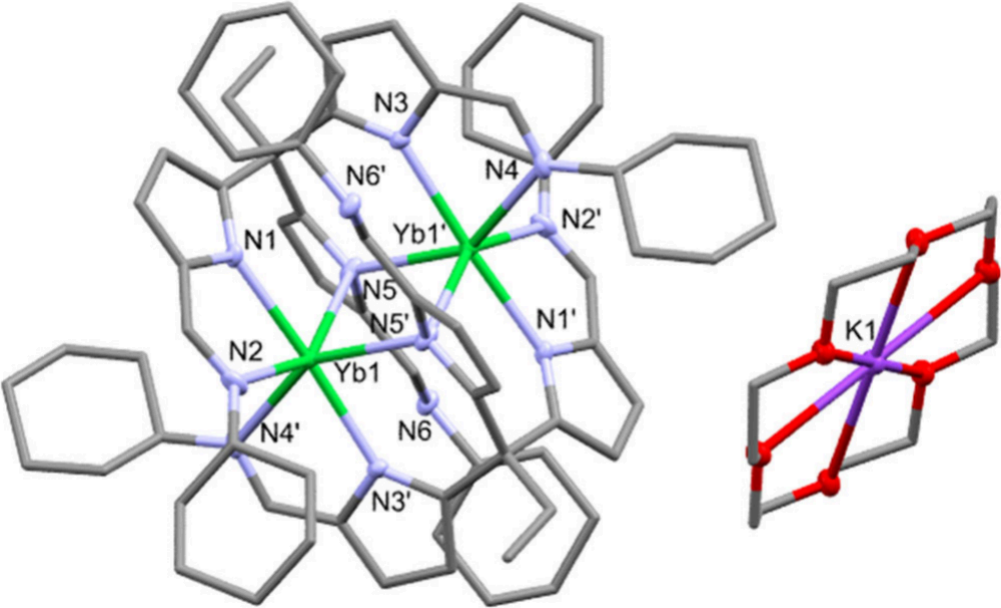
The X-ray crystal structure of [K(18-c-6)][Yb(L)]_2_, **2**. For clarity, all hydrogen atoms and noncoordinating pyridine solvent are omitted, and any displacement ellipsoids are drawn at 50% probability. Selected bond distances and angles are summarized in Table SI 1. [Color code: Yb, green; K, purple; N, blue; O, red; C, gray.]

The reduced dimer **2** is more compact than in **1**, with an Yb**···**Yb distance of 3.5807(3) Å. This Yb**···**Yb distance is shorter than many two-center, one-electron bond distances reported for homobimetallic lanthanide complexes (range of 3.3992(6)–3.960(1) Å) but is longer than the Pyykkö covalent distance (3.40 Å).^6,7,11^ This short Yb**···**Yb distance could be an indicator of a strong metal–metal interaction and is within a reasonable range for an Yb–Yb single-electron bond. The centrosymmetric structure of the anion of **2** means that the coordination environment at each metal is identical. Unusually, no solvent is coordinated to the potassium-18-crown-6 center, which, instead, shows C–H to K interactions from the ligand cyclohexyl groups (see Figures SI 3 and SI 4 and Table SI 2). An essentially identical centrosymmetric solid-state structure [K([2.2.2]crypt)][Yb(L)]_2_ (**5**) is obtained using [2.2.2]crypt in place of 18-crown-6 (Figure SI 5 and Table SI 3). The X-ray structures of **2** and **5** do not categorically confirm the Yb valencies. However, the centrosymmetric nature of the structure of **2** is supported by an unsuccessful attempt to solve and refine the X-ray model in the noncentrosymmetric space group *P*1. Also, disorder in the structures of **2** and **5** is unlikely, because no residual peaks are seen in the difference Fourier maps and no unusual displacement ellipsoids are seen.

The addition of 2 equiv of potassium graphite and 18-crown-6 to a solution **1** in THF gives a dark brown solution of K{Yb(py)(L)} **3** that displays diamagnetic ^1^H and ^13^C{^1^H} NMR spectra consistent with the ligand adopting *C*_3_-symmetry (see Figures SI 6 and SI 7). These spectra suggest that metal reduction to Yb(II) has occurred and that **3** is monomeric; unfortunately, all attempts to crystallize **3** were unsuccessful. However, it is possible to prepare monomeric Y(py)(L) **4**, which provides support for the nuclearity and symmetry of **3** (ionic radius for seven-coordinated Yb^2+^ = 1.08 Å and Y^3+^ = 0.96 Å).^12^ The X-ray crystal structure of **4** shows a monomeric complex of approximate *C*_3_-symmetry (Figure SI 8) and its NMR spectra (see Figures SI 9–SI 11) display resonances consistent with this symmetry and are similar to those of **3**.

To better understand the electronic structure of **2**, density functional theory (DFT) calculations were undertaken. Three possible scenarios exist: (i) reduction of a single Yb^3+^ center resulting in localized Yb^2+^/Yb^3+^ cations; (ii) single-electron reduction of the ligand resulting in three paramagnetic centers, Yb^3+^/L^• –^/Yb^3+^; (iii) equal sharing of the single electron by both Yb centers, i.e., Yb^2.5+^/Yb^2.5+^. Scenario (i) is improbable, because no convergence in DFT calculations is seen, likely due to the rigidity of the structure causing retention of centrosymmetry. DFT calculations on scenario (ii) result in the generation of a radical center on the ligand and a strong ferromagnetic interaction between this radical and the Yb^3+^ centers (see Figures SI 13–SI 15).^13^ However, this scenario can be discounted as SQUID magnetic data for **2** (Figure SI 12) support the presence of a single unpaired *f*-electron only.

For scenario (iii), DFT-based spin density analysis on **2** suggests almost equal sharing of the additional electron between both Yb centers (Figure 2a). This observation is unlike other dinuclear Gd, Tb, and Dy lanthanide complexes that form direct Ln–Ln bonds through the occupation of a diffuse 5*d* orbital.^6^ To probe the electronic structure further, Quantum Theory of Atoms in Molecules (QTAIM)^14^ was carried out on **2**, which shows no bond-center point between the two Yb centers, suggesting the absence of an Yb–Yb bond (see Figures 2b and SI 16). This is further supported by the Wiberg Bond Index of 0.17, which implies minimal Yb–Yb bonding.^15^

**Figure 2:**
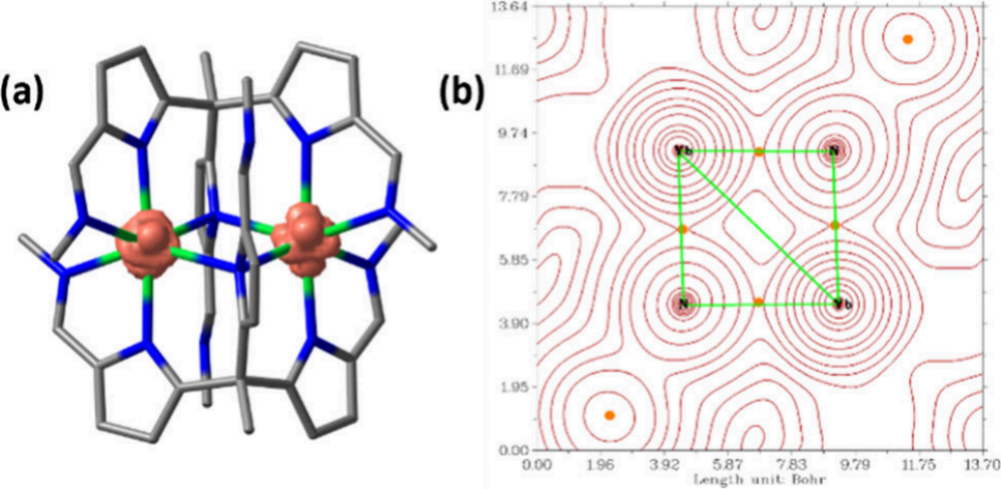
(a) DFT computed spin density plot for **2**. The isodensity surface corresponds to 0.03 e bohr^–3^. (b) DFT-QTAIM computed contour map for **2**. Orange dots represent the bond center points (BCPs).

Electronic absorption spectroscopy was carried out on compounds **1** and **2** (see Figures 3a and SI 17). Compound **1** is bright yellow, and **2** is dark green/brown, and both feature two sharp, low-intensity (ε = 10–50 M^–1^ cm^–1^) 4*f* → 4*f* transitions in the near-IR region (900–1000 nm). These absorptions are similar to those seen for the mixed-valence complex [Yb_2_(NP(pip)_3_)_5_] ([NP(pip)_3_] = tri(piperidinyl)imidophosphorane), that were assigned as the 4*f*^13^ [^2^F_7/2_ → ^2^F_5/2_] transitions.^4^ Importantly, a very broad, low-intensity absorbance (ε = 258 M^–1^ cm^–1^) is seen at ca. 600 nm in the spectrum of **2** that is not present in **1**. A very similar feature was seen for [Yb_2_(NP(pip)_3_)_5_] and was assigned as a 4*f*^14^ → 4*f*^13^ intervalence charge transfer (IVCT). A similar assignment for **2** is further supported by the calculated Γ parameter of 0.995, suggesting that **2** is a Class III mixed-valence compound.^1^ In both of the spectra for **1** and **2**, an absorbance at 334 nm (ε = 127 600–132 800 M^–1^ cm^–1^) is evident, which is likely a ligand-based, π → π* transition.

**Figure 3:**
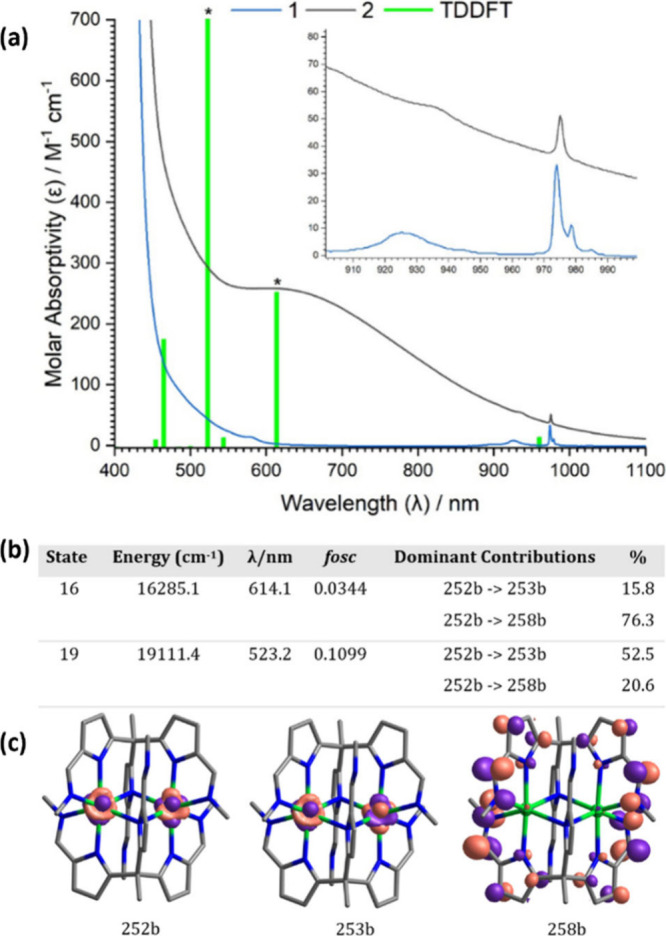
(a) UV–vis–NIR absorption spectrum of **1** (blue) and **2** (black) (THF, 400–1100 nm). The results of time-dependent density functional theory (TDDFT) calculations of the 4*f* → 4*f* transition energy of **2** are overlaid (green bars), and selected calculated absorption spectra by TD-camB3LYP are shown with asterisks that represent 4*f* → 4*f* IVCT electronic transitions. (b) Absorptions with nonzero oscillator strength for 4*f*→ 4*f* transitions are summarized in the table (*fosc* = oscillator strength). (c) The orbitals involved in the 4*f***→** 4*f* transitions. Only states involved in 4*f* → 4*f* transitions and that have more than 10% 4*f* → 4*f* contributions are shown.

To better assign the electronic transitions associated with the UV–vis–NIR spectrum of **2**, time-dependent DFT (TDDFT) and SA-CASSCF/NEVPT2+SOC calculations were undertaken; these computational methods are known to reproduce the experimental absorption spectra well.^4^ The calculated absorption spectra by TD-camB3LYP for **2** predict the 4*f* → 4*f* IVCT transitions at 614.1 and 523.3 nm (Figure 3b). The CAS calculations, which consider only metal-based 4*f* electrons, further predict a weaker IVCT band at 598.6 nm (*fsoc* = 0.0165). As such, these calculations support the classification of **2** as a Class III mixed-valence complex that exhibits intervalence charge transfer.

In summary, a dinuclear ytterbium(III) complex has been synthesized and characterized and its single-electron reduction has resulted in a new mixed-valence complex with two Yb^2.5+^ oxidation states, assigned as Robin–Day Class III, with no metal–metal bonding. To the best of our knowledge, this is the first Robin–Day Class III, Yb-based mixed-valence molecule reported and the second among all Yb-based Robin–Day Classes I–III to show IVCT phenomenon.

There are limited examples of molecular mixed-valence lanthanide compounds, and even fewer displaying IVCT. This new example of a two-metal one-electron reduction reaction helps expand our understanding of the different electronic structures available to the lanthanides, and contrasts with that seen in two-center, one-electron M–M bond formation and localized mixed-valence Yb^2+^–Yb^3+^ IVCT previously seen. It is anticipated that further insight into electron transfer processes may be gained by exploring the electronic structures of other homodinuclear and heterodinuclear lanthanide complexes of this rigid polydentate framework, especially for those with relatively accessible 2+ oxidation states such as Sm and Eu.

## Supporting Information Available

Experimental procedures, ^1^H and ^13^C{^1^H} NMR spectra, X-ray structures and bond distances, magnetic susceptibility and magnetization measurements, computational details, additional UV–vis–NIR spectra, TD-B3LYP computed transition probabilities, cyclic voltammogram and Randles–Ševčik plot, crystallographic details, DTF-optimized *xyz* coordinates, inputs for TD-camB3LYP and CASSCF calculations (PDF)
