# Supplementary material for: A Delocalized Mixed-Valence Dinuclear Ytterbium Complex That Displays Intervalence Charge Transfer
Source: J Am Chem Soc. 2024 Oct 14;146(42):28658–62. doi: 10.1021/jacs.4c12188 (PMC11503763; doi:10.1021/jacs.4c12188)
Supplement: Supplementary file 1 — ja4c12188_si_001.pdf [file ja4c12188_si_001.pdf]

A delocalized mixed-valence dinuclear ytterbium complex that displays intervalence charge transfer.

Tom J. N. Obey, Mukesh K. Singh, Angelos B. Canaj, Gary S. Nichol, Euan K. Brechin and Jason B. Love*.

Contents

1. General Procedures 1
2. Synthesis 3
H_3L 3
1 3
2 4
3 4
4 5
3. Magnetic Measurements 13
4. Computational Details 14
5. Electrochemistry 21
6. Crystallographic details 24
7. DFT optimized xyz coordinates for complex 2 (without K-18-Crown-6) 28
8. Input for TD-camB3LYP calculation. 30
9. Input for CASSCF calculation 30
10. References 32

1. General Procedures

The synthesis of all air- and moisture-sensitive compounds was carried out either using standard Schlenk techniques under an atmosphere of dry argon or in MBraun gloveboxes under an atmosphere of dry dinitrogen. Vacuum atmospheres and MBraun gloveboxes were used to store and handle the air- and moisture-sensitive compounds. The solvents d_5-pyridine and d_6-benzene were refluxed over potassium metal overnight, freeze-pump-thaw degassed three times and distilled under reduced pressure prior to use. All glassware and cannulae were stored in a 160°C oven overnight before use. Dry solvents were collected from a solvent purification system (Innovation Technologies) and stored in Teflon-tapped ampules over pre-dried 4 Å molecular sieves. Tetrabutylammonium hexafluorophasphate, [^{n}Bu$_4$N][PF$_6$], was recrystallized twice from ethanol and dried under reduced pressure before use. 5,5′,5′′-triformyl-2,2′,2′′-tripyrrolylpropane, ytterbium tris(bis(trimethylsilyl)amide), yttrium tris(bis(trimethylsilyl)amide) and gadolinium tris(bis(trimethylsilyl)amide) were synthesised following literature procedures.[1] 18-Crown-6 and [2.2.2]cryptand were sublimed prior to use. All other chemicals were used as received without further purification unless specified.

^{1}H and ^{13}C{^{1}H} NMR spectra were recorded on a Bruker AVA400 spectrometer at 399.90 MHz and 100.61 MHz, Bruker AVA500 spectrometer at 500.12 MHz and 125.76 MHz, or Bruker AVA600 spectrometer at 599.81 MHz and 151.05 MHz, respectively. ^{29}Si{^{1}H} NMR spectra were recorded on a Bruker PRO500 spectrometer at 470.59 MHz. All chemical shifts are reported in parts per million (ppm). ^{1}H and ^{13}C{^{1}H} NMR data were referenced to residual solvent signals. Spectra were recorded at 298 K and data were processed using MestReNova 14.0.0. Single-crystal X-ray diffraction data were

collected on an Oxford Diffraction Excalibur diffractometer using graphite monochromated Mo K_{α} radiation equipped with an Eos charge-coupled device detector (λ = 0.71073 Å) at 120 K, or on a Supernova, Dual, Cu at zero Atlas diffractometer using Cu K_{α} radiation (λ = 1.5418 Å) at 120 K. All structures were solved using ShelXT direct methods and refined using a full-matrix least-squares refinement on $|F|^2$ using ShelXL.[2] All programs were used within the Olex2 suite.[3] Non-hydrogen atoms were refined with anisotropic displacement parameters and hydrogen atoms were constrained to parent atoms and refined using a riding model. Structures were analyzed and illustrated in Mercury 4.3.1.[4] Mass spectrometry was recorded using a 12T Bruker SolariX with APPI source with a portable glovebox flushed with argon surrounding the injection port. UV-vis-NIR spectroscopy was performed using a quartz cuvette with a path length of 10 mm on a NanoDrop 2000c photospectrometer scanning between 1100 and 300 nm. Cyclic voltammetry measurements were made using an Autolab 302 potentiostat and data processed in NOVA 2.1.5. All experiments were carried out at room temperature in a glovebox under an atmosphere of dry dinitrogen. A platinum working electrode, platinum counter electrode and silver wire pseudo-reference electrode were used with a 0.1 M solution of $[^{n}Bu_4N][PF_6]$ in THF. All experiments were referenced against $[FeCp_2]^{+/0}$ ($E_{1/2}$, $[FeCp_2]^{+/0}$ = 0.0V). Elemental analysis was carried out by the London Metropolitan University and measured in duplicate. Magnetic susceptibility and magnetization data were collected on a freshly prepared polycrystalline sample (typical mass of ~13 mg) on a Quantum Design Dynacool PPMS equipped with a 9 T magnet in the temperature range 300 - 2K. The samples were prepared and weighed in an Argon Glovebox (MBraun) and then added in Quantum Design VSM Powder Sample Holders (P125E). The holder with the sample was transferred to a PPMS brass half-tube sample holder and sealed in a Schlenk tube before being brought outside the glovebox and inside the PPMS. Diamagnetic corrections from the holders were applied. Diamagnetic corrections were also applied to the observed paramagnetic susceptibilities using Pascal's constants.

2. Synthesis

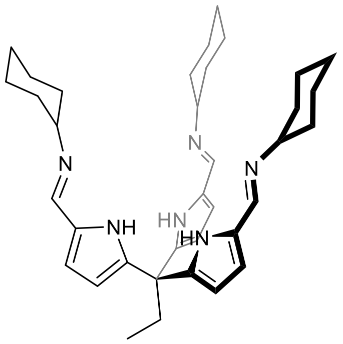

H_3L: The synthesis of **$H_3L(H_2O)$** was adapted from the literatures procedures.[5] Cyclohexylamine (10.8 mL, 94.19 mmol, 3.3 eq.) was added dropwise to a solution of tris(5,5′,5′′-formyl-2,2′,2′′-pyrrolyl)propane (9.27 g, 28.67 mmol) in THF (100 mL) at 0 °C. The mixture was allowed to warm to room temperature before being stirred at 60 °C for 16 hours. After which the solvent was removed under reduced pressure to obtain an off-white solid. This was washed with acetonitrile to obtain the product, **$H_3L(H_2O)$**, as a colorless solid (12.92 g, 22.09 mmol, 76%). The H_2O was removed by dissolving **$H_3L(H_2O)$** (9.57 g, 0.017 mol) in dry toluene (100 mL) under an argon atmosphere followed by the addition of trimethylsilyl chloride (7 mL, 0.055 mol, 3.2 eq.) was added. A pale brown precipitate formed immediately. The solid was isolated by filtration and washed with dry toluene before suspension in toluene (100 mL). A solution of 1,4-diazabicyclo[2.2.2]octane (DABCO) (1.89 g, 0.017 mol) in dry toluene (20 mL) was added to the suspension and the mixture stirred for 16 hours. The mixture was filtered under argon and the solvent removed from the yellow filtrate under reduced pressure to afford a pale brown solid. This solid was washed with acetonitrile to give the pure product as a colorless solid (4.78 g, 8.43 mmol, 50%). ^{1}H NMR (500 MHz, d_5-pyridine): δ_H 10.59 (br. s, 3H, pyrrolyl-NH), 8.05 (s, 3H, imino-CH), 6.52 (d, J = 3.6 Hz, 3H, pyrrolyl-CH), 6.26 (d, J = 3.6 Hz, 3H, pyrrolyl-CH), 2.99 (m, 3H, cyclohexyl-NCH), 2.69 (q, J = 7.4, 6.9 Hz, 2H, ethyl-CH_2) 1.68 – 1.62 (m, 12H, cyclohexyl-CH), 1.55 – 1.49 (m, 3H, cyclohexyl-CH), 1.42 (m, 6H, cyclohexyl-CH), 1.29 – 1.18 (m, 6H, cyclohexyl-CH), 1.09 (m, 3H, cyclohexyl-CH), 0.99 – 0.91 (t, 3H, ethyl-CH_3). ^{13}C{^{1}H} NMR (126 MHz, d_5-pyridine): δ_C 140.04, 131.80, 114.09, 109.65, 69.69, 48.30, 47.23, 35.37, 25.35, 10.55. APPI-MS: Found, 567.41764 m/z; [$C_{36}H_{50}N_6$](+H^+) requires, 567.41697 m/z (mass error = 1.18 ppm).

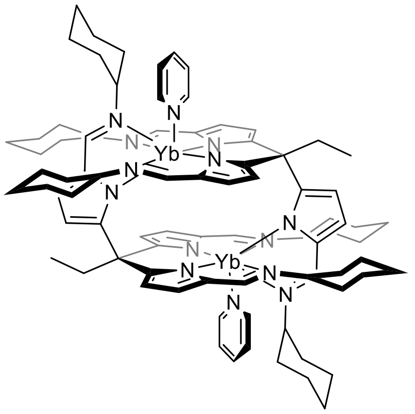

1: A solution of **H_3L** (0.890 g, 1.570 mmol) in dry toluene (30 mL) was added to a stirred solution of $Yb(N(SiMe_3)_2)_3$ (1.027 g, 1.570 mmol, 1 eq.). The mixture was stirred at room temperature for 2 hours after which a pale yellow precipitated formed. The precipitate was removed by filtration and the solvent removed from the filtrate under reduced pressure. The yellow solid that was obtained was dissolved in dry pyridine and layered with hexane. The product, **1**, was obtained as yellow block-shaped crystals after several days (0.830 g, 1.017 mmol, 65%). ^{1}H NMR (500 MHz, d_8-THF): δ_H 75.18, 57.90, 49.83, 34.28, 29.32, 17.51, 14.97, 13.34, 10.25, 8.54, 7.64, 7.24, 1.29, 0.89, 0.10, -1.44, -4.00, -6.04, -12.32, -14.84, -19.49, -20.23, -27.97, -30.49, -31.37, -33.35, -46.92, -47.46. APPI-MS: Found,

1475.67594 m/z; [$Yb_2C_{72}H_{94}N_{12}$](+H^+) requires, 1475.67633 m/z (mass error = 10.19 ppm) . Found: C, 58.63; H, 6.74; N, 11.47; %. [$Yb_2C_{72}H_{94}N_{12}$] requires: C, 58.68; H, 6.43; N, 11.41; %.

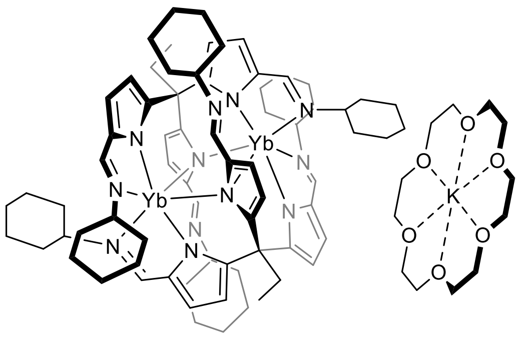

2: 18-Crown-6 (27 mg, 0.102 mmol) was added to a stirred solution of **1** (150 mg, 0.102 mmol, 1 eq.) in dry THF (10 mL). Potassium graphite (15 mg, 0.111 mmol, 1.1 eq.) was added to the yellow solution and the mixture stirred for 16 hours. The graphite was removed by filtration and the solvent removed from the now dark brown filtrate. The resulting brown solid was dissolved in the minimum quantity of dry pyridine and layered with hexane. The product, **2**, was obtained as dark brown block crystals after several days (63 mg, 0.035 mmol, 35%). No resonances in ^{1}H NMR spectrum were observed. APPI-MS: Found, 1475.67633 m/z; [$C_{72}H_{94}N_{12}Yb_2$](+H^+) requires, 1475.67633 m/z (mass error = 13.25 ppm). Found: C, 57.67; H, 6.55; N, 10.00; %. [$C_{84}HKN_{12}O_6Yb_2(C_5H_5N)$] requires: C, 57.59; H, 6.68; N, 9.81; %.

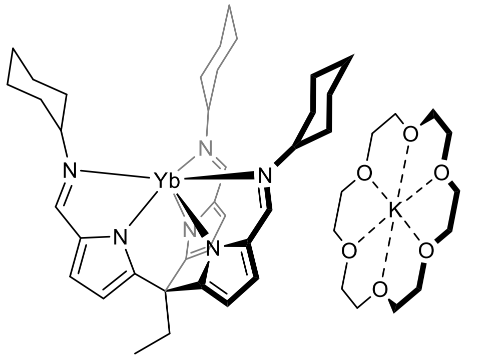

3: 18-Crown-6 (8 mg, 0.030 mmol, 2 eq) was added to a stirred solution of **1** (20 mg, 0.014 mmol) in dry THF (5 mL). Potassium graphite (4 mg, 0.030 mmol, 2 eq) was added at −20°C and the stirred yellow solution became dark brown after several minutes. This mixture was allowed to warm to room temperature and stirred for 16 hours before the graphite was removed by filtration and the solvent removed under reduced pressure. The dark brown solid was washed with hexane to give the potential product [K(18c6)][Yb^{II}(L)] **3** (8 mg, 0.08 mmol, 55%). ^{1}H NMR (500 MHz, d_5-pyridine): δ_H 8.14 (s, 3H, imino-CH), 6.58 (d, *J* = 3.2 Hz, 3H, pyrrolyl-CH), 6.54 (d, *J* = 3.1 Hz, 3H, pyrrolyl-CH), 3.56 (s, 24H, 18-crown-6), 3.21 (q, *J* = 7.2 Hz, 2H, ethyl-CH_2), 3.14 (m, 3H, cyclohexyl-NCH), 2.01 (d, *J* = 12.9 Hz, 6H, cyclohexyl-CH), 1.93 (t, *J* = 7.3 Hz, 3H, ethyl-CH_3), 1.86 – 1.80 (m, 6H, cyclohexyl-CH), 1.73 (q, *J* = 12.4 Hz, 9H, cyclohexyl-CH), 1.39 – 1.22 (m, 9H, cyclohexyl-CH). $^{13}C\{^1H\}$ NMR (151 MHz, d_5-pyridine): δ_C 159.73, 159.02, 137.35, 135.98, 135.78, 112.85, 105.85, 70.99, 67.22, 52.61, 37.11, 26.59, 12.94. APPI-MS: Found, 738.33615 m/z; [$YbC_{36}H_{47}N_6$](+H^+) requires, 738.33295 m/z (mass error = 5.13 ppm).

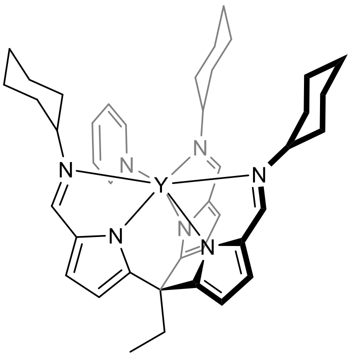

4: **H_3L** (500 mg, 0.883 mmol) was dissolved in dry toluene (20 mL). $Y(N(SiMe_3)_2)_3$ (503 mg, 0.883 mmol, 1 eq.) in toluene (10 mL) was added to the solution and stirred at room temperature for 1 hour. The solid precipitate that formed was filtered and washed with toluene and hexane giving the product, **4**, as yellow needle shaped crystals after recrystallization from a pyridine solution layered with hexane (275 mg, 0.421 mmol, 48%). ^{1}H NMR (500 MHz, pyridine-*d*$_5$): δ_H 7.86 (s, 3H, imino-CH), 6.43 (d, *J* = 1.5 Hz, 3H, pyrrolyl-CH), 6.42 (d, *J* = 1.5 Hz, 3H, pyrrolyl-CH), 3.31 (m, 3H, cyclohexyl-NCH), 2.93 (q, *J* = 7.4 Hz, 2H, ethyl-CH_3), 2.01 (d, *J* = 11.8 Hz, 6H, cyclohexyl-CH), 1.82 (d, *J* = 13.0 Hz, 6H, cyclohexyl-CH), 1.76 (t, 3H, ethyl-CH_2), 1.69 (d, *J* = 13.0 Hz, 6H, cyclohexyl-CH), 1.45 (q, *J* = 12.0 Hz, 6H, cyclohexyl-CH), 1.34 (q, *J* = 12.8 Hz, 6H, cyclohexyl-CH). ^{13}C{^{1}H} NMR (101 MHz, pyridine-*d*$_5$): δ_C 160.68, 160.29, 136.78, 115.53, 107.75, 63.10, 52.54, 35.80, 26.42, 26.14, 24.85, 11.88. APPI-MS: Found, 653.30162 m/z; $[YC_{36}H_{47}N_6](H^+)$ requires, 653.29934 (mass error = 2.91 ppm). Found: C, 66.29; H, 7.42; N, 12.62; %. $[YC_{36}H_{47}N_6]$ requires: C, 66.25; H, 7.26; N, 12.88; %.

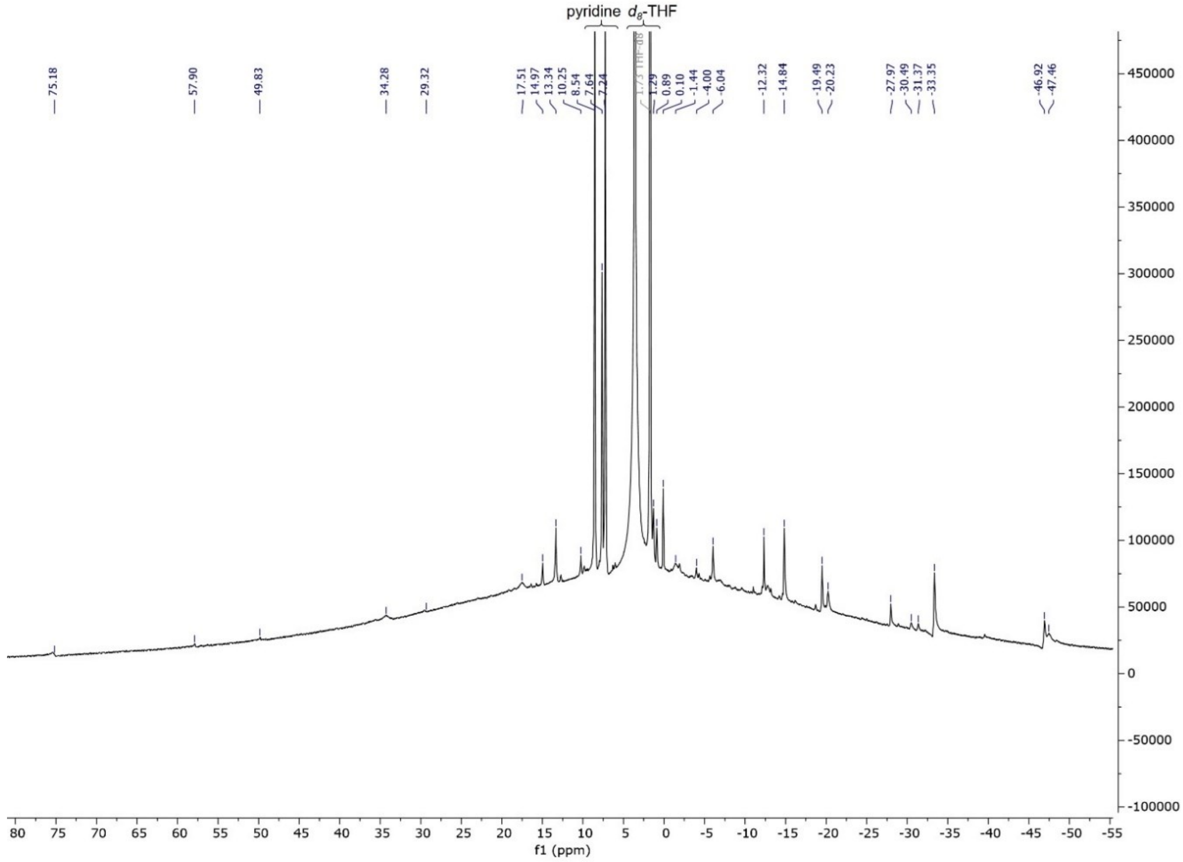

Figure SI 1. ^{1}H NMR spectrum of **1** in *d*$_8$-THF.

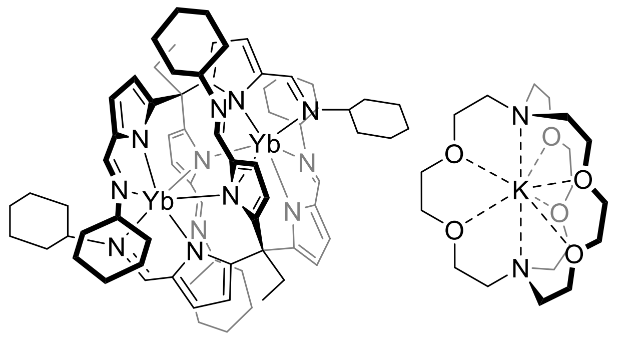

5: [2,2,2]Cryptand (25 mg, 0.066 mmol, 1.8 eq.) was added to a stirred solution of **1** (55 mg, 0.037 mmol) in dry THF (5 mL). Potassium graphite (9 mg, 0.067 mmol, 1.8 eq.) was added to the yellow solution and the mixture stirred for 16 hours. The graphite was removed by filtration and the solvent removed from the now dark brown filtrate. The resulting brown solid was dissolved in the minimum quantity of dry pyridine and layered with hexane. The product, **5**, was obtained as dark brown block shaped crystals after several days, however no yield was calculated as only a single crystal was isolated. No resonances in ^{1}H NMR spectrum were observed.

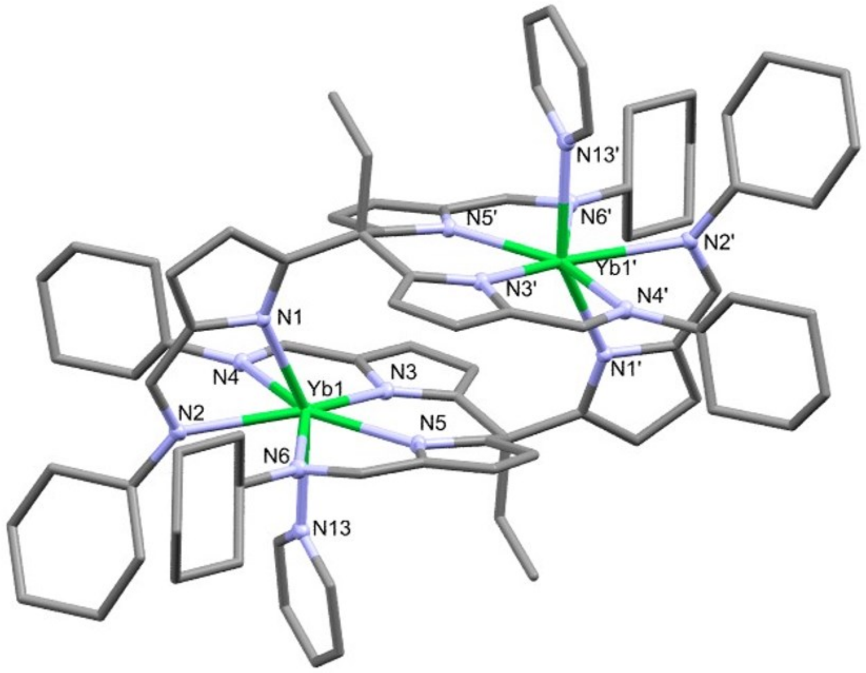

Figure SI 2. X-ray crystal structure of $[Yb(L)(py)]_2$, **1**. For clarity, all hydrogen atoms are omitted and any displacement ellipsoids are drawn at 50% probability. Color code: Yb, green; N, blue; C, grey.

Distance		1	Distance		2
N_{imino}	Yb1-N2	2.475(1)	N_{imino}	Yb1-N2	2.442(2)
	Yb1-N4	2.568(1)		Yb1-N6’	2.433(2)
	Yb1-N6	2.518(1)			
$N_{pyrrolyl}$	Yb1-N1	2.349(1)	$N_{pyrrolyl}$	Yb1-N1	2.361(2)
	Yb1-N3	2.338(1)		Yb1-N3	2.530(2)
	Yb1-N5	2.374(1)		Yb1-N3’	2.497(2)
				Yb1-N5’	2.358(2)
	Yb1···Yb1’	5.6214(5)		Yb1···Yb1’	3.5807(3)

Table SI 1. Selected bond distances (Å) for the dimeric Yb^{3+} complex, **1**, and the mixed-valence complex, **2**.

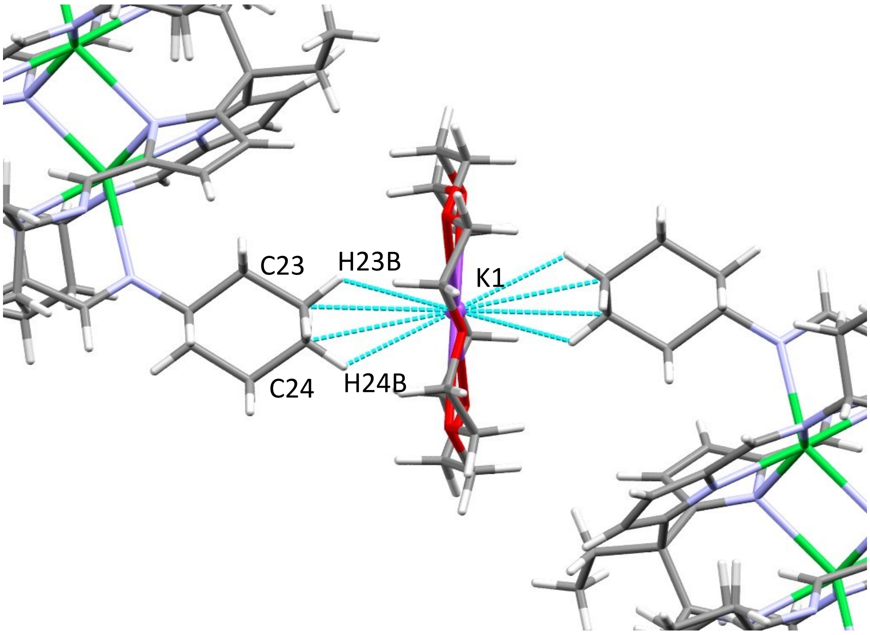

Figure SI 3. The short contacts showing C-H···K interaction between the potassium cation and the ligand cyclohexyl groups in complex **2**. Color code: Yb, green; K, purple; N, blue; O, red; C, grey.

Distance	Å
K1-C23	3.350(2)
K1-C24	3.470(3)
K1-H23B	2.77143(5)
K1-H24	2.85013(5)

Table SI 2. Intermolecular distances between the potassium cation and the cyclohexyl groups in complex **2**.

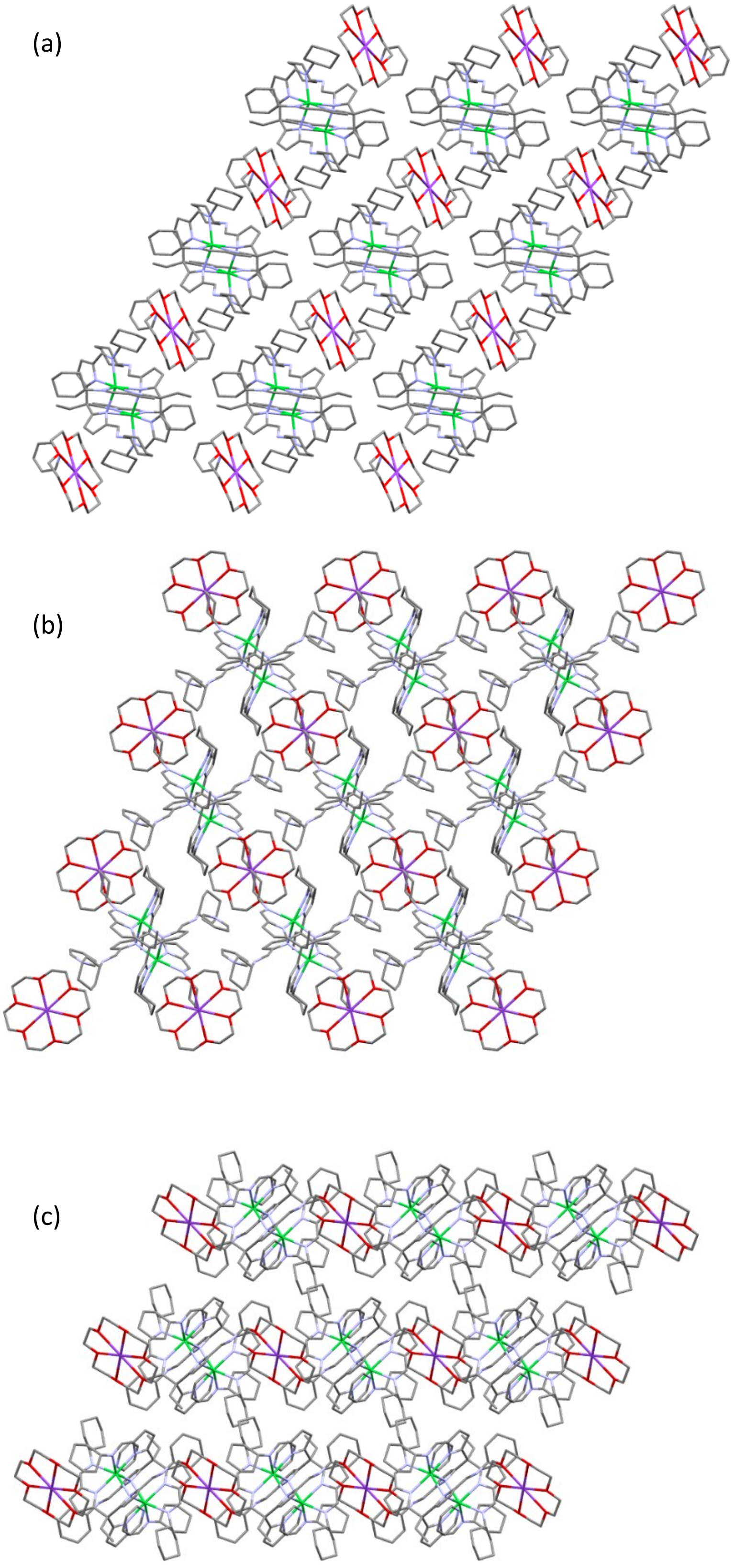

Figure SI 4. The packing in the X-ray structure of **2** displayed along the a (TOP), b (MIDDLE) and c (BOTTOM) axes.

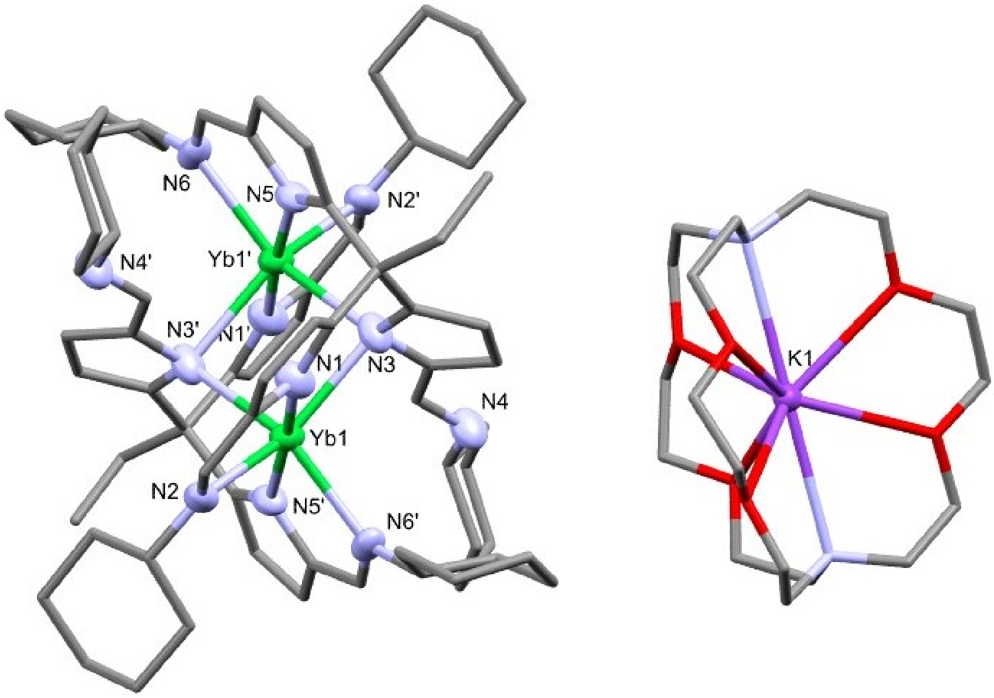

Figure SI 5. The X-ray crystal structure of the complex [K([2.2.2]crypt)][Yb(L)]$_2$ (**5**). For clarity, all hydrogen atoms are omitted and any displacement ellipsoids are drawn at 50% probability. Selected bond distances and angles for **2** and **5** are compared in Table SI 3. Color code: Yb, green; K, purple; N, blue; O, red; C, grey.

	Distance	2	5
N_{imino}	Yb1-N2	2.442(2)	2.429(6)
	Yb1-N6′	2.433(2)	2.440(4)
$N_{pyrrolyl}$	Yb1-N1	2.361(2)	2.354(6)
	Yb1-N3	2.530(2)	2.526(5)
	Yb1-N3′	2.497(2)	2.503(6)
	Yb1-N5′	2.358(2)	2.352(5)
	Yb1···Yb1′	3.5807(3)	3.5961(7)

Table SI 3. Selected bond distances (Å) for the dimeric Yb complexes **2** and **5**.

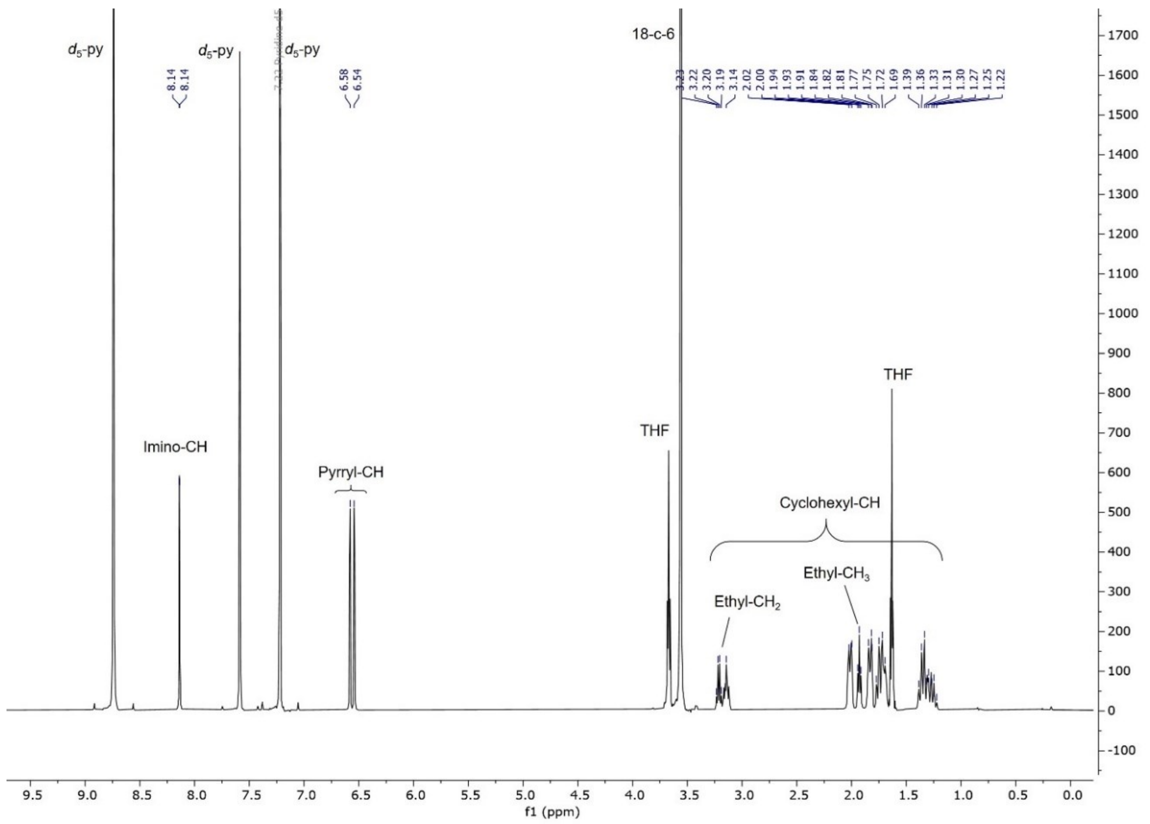

Figure SI 6. ^{1}H NMR spectrum of the Yb(II) complex, **3**.

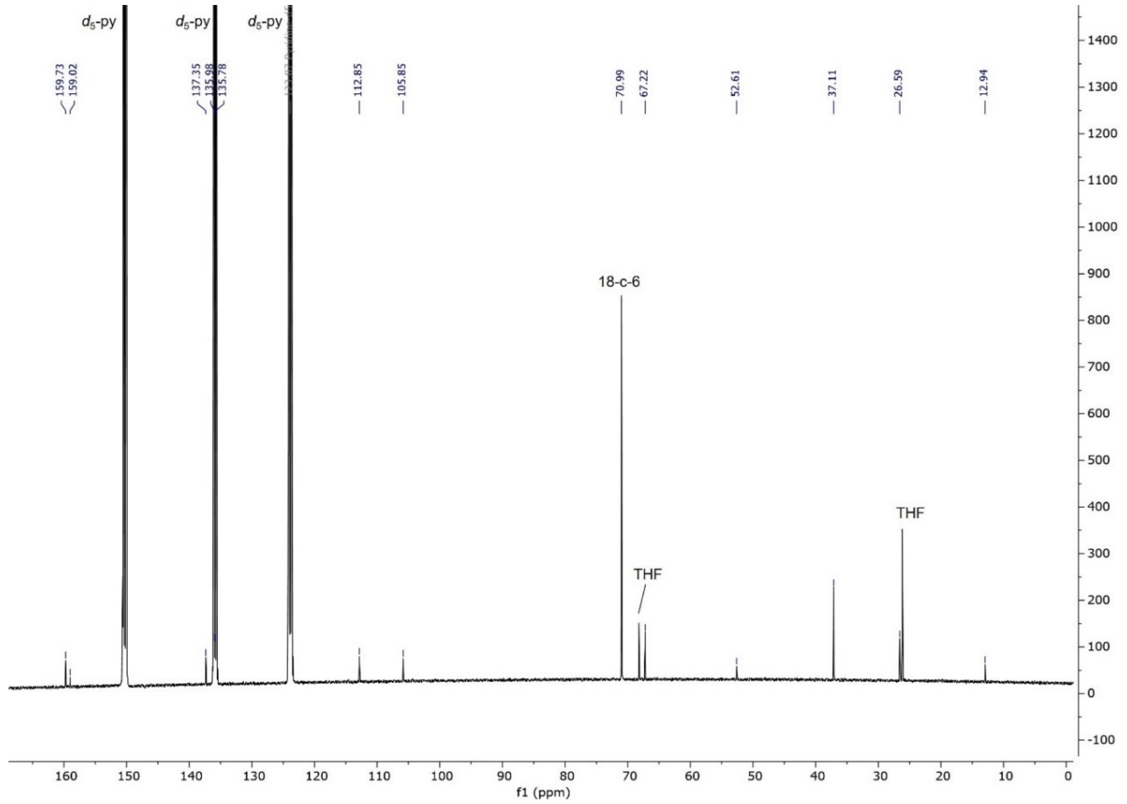

Figure SI 7. ^{13}C{^{1}H} NMR spectrum of the Yb(II) complex, **3**.

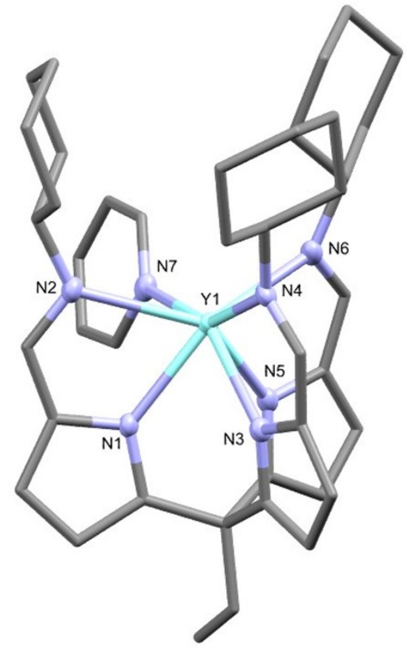

Figure SI 8. The X-ray crystal structure of the monomeric Y complex **4**. For clarity, all hydrogen atoms are omitted and any displacement ellipsoids are drawn at 50% probability. Selected bond distances (Å): Y1-N1 2.303(2), Y1-N2 2.563(2), Y1-N3 2.334(2), Y1-N4 2.598(3), Y1-N5 2.316(2), Y1-N6 2.523(2), Y1-N7 2.632(3). Color code: Y, light blue; N, blue; C, grey.

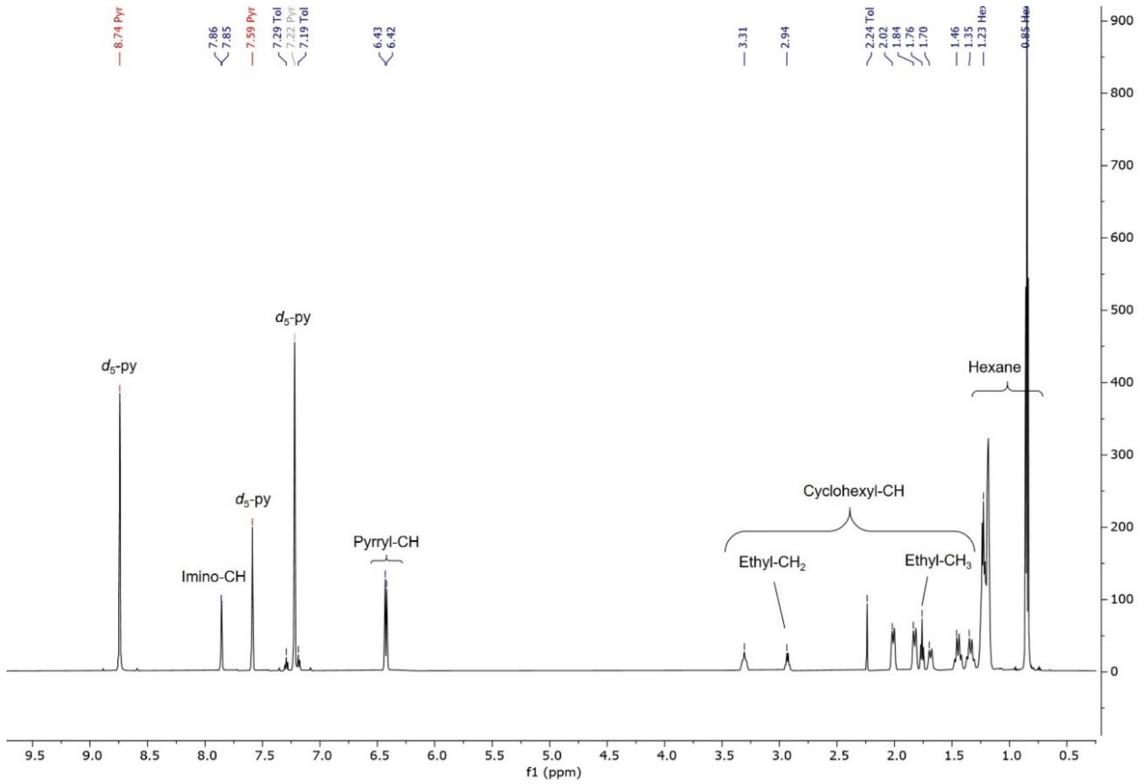

Figure SI 9. ^{1}H NMR spectrum of **4** in d_5-pyridine.

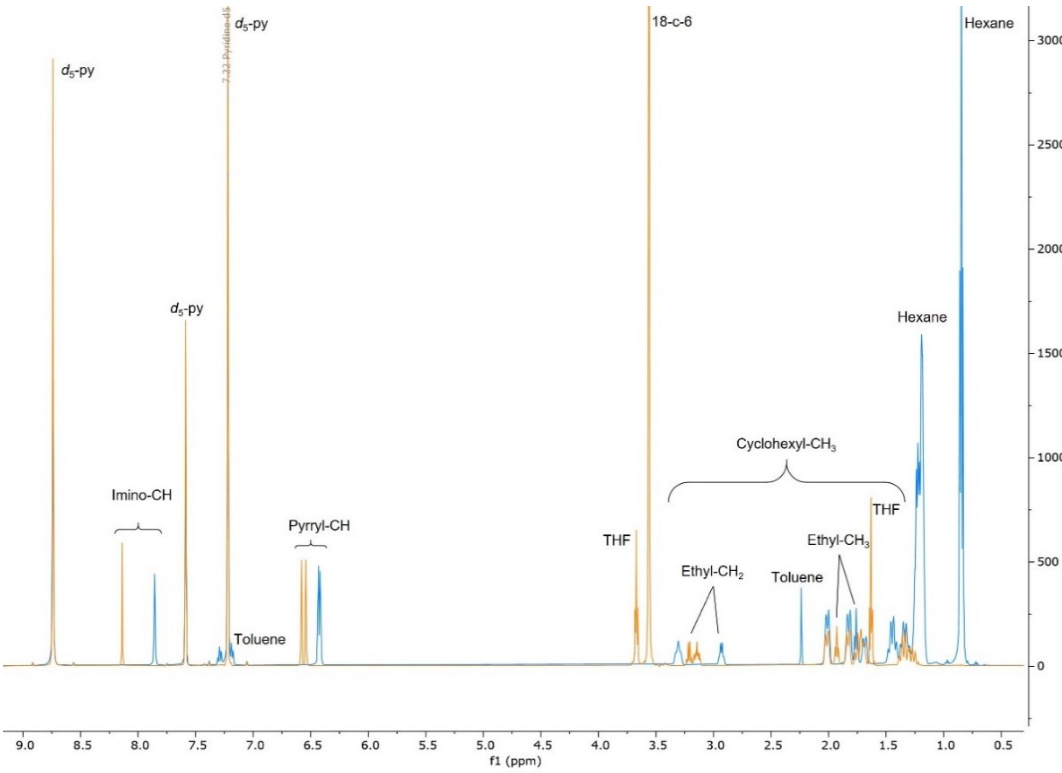

Figure SI 10. $^{13}C\{^{1}H\}$ NMR spectrum of **4** in *d*5-pyridine.

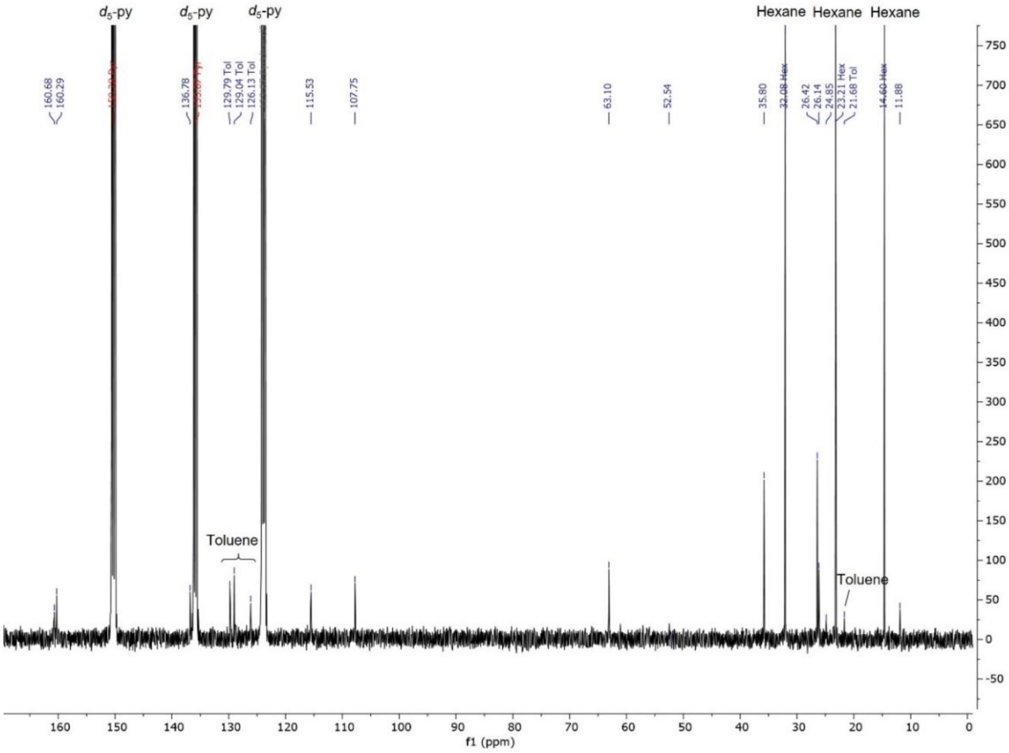

Figure SI 11. Comparison of the ^{1}H NMR spectra of **3** (orange) and **4** (light blue) in *d*5-pyridine. As expected, for complex **3** which has an Yb^{2+} oxidation state, more deshielding of proton peaks for pyrrolyl-CH and imino-CH are seen compared with complex **4** which comprises Y^{3+}.

3. Magnetic Measurements

Variable temperature magnetic susceptibility and magnetization measurements were undertaken for **2** in order to explore the possible electronic and magnetic features of **2**, where the additional electron can either reduce one of the Yb^{3+} centers to Yb^{2+}, become a ligand-based radical bridging both Yb^{3+} ions, or be shared between both Yb^{3+} ions, resulting in two $Yb^{2.5+}$ ions.

Magnetic susceptibility (χ) and magnetization (M) measurements were performed on a polycrystalline sample of **2** in the ranges T = 300 - 2 K, H = 0.1 T, and T = 2-10K, H = 0.5-9.0 T, respectively. The experimental $\chi_M T$ value of 2.55 cm^3 K mol^{-1} at T = 300 K is very close to the expected value of 2.57 cm^3 K mol^{-1} for a single non-interacting Yb^{3+} ion ($^2F_{7/2}$, S = 1/2, L = 3, J = 7/2) (Figure 5, SI 12a). As the temperature is lowered, the value of $\chi_M T$ decreases, reaching a value of 1.67 cm^3 K mol^{-1} at 10 K before decreasing more rapidly to a value of 1.55 cm^3 K mol^{-1} at 2 K. The observed decrease of $\chi_M T$ upon cooling is consistent with the thermal depopulation of the higher-energy m_J levels.

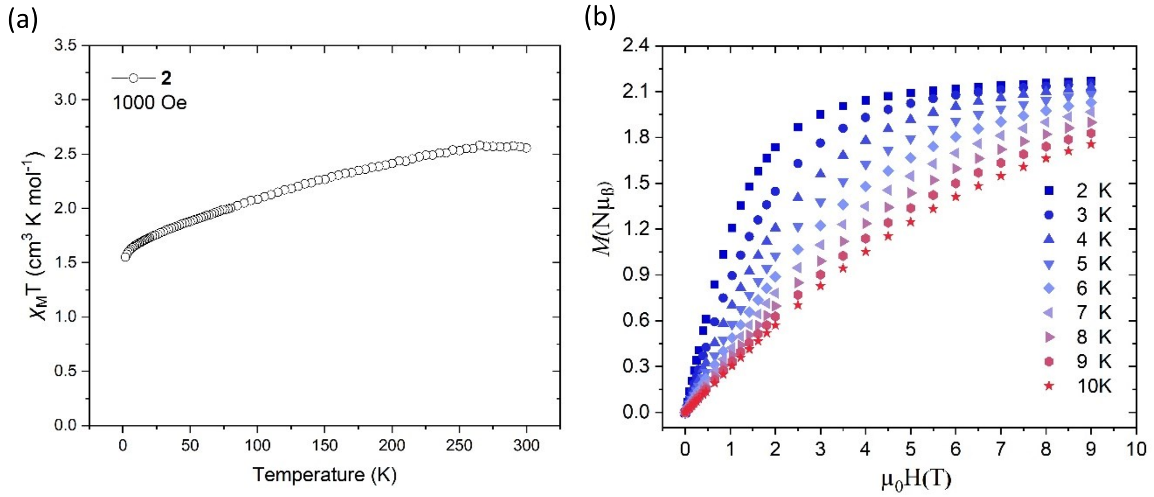

Figure SI 12. Plot of $\chi_M T$ versus T (a) and *magnetization* versus *field* (b) for **2**.

This magnetic susceptibility measurement confirms the presence of a single unpaired electron, ruling out the possibility of two Yb^{3+} ions with a ligand-based radical. This suggests the presence of either a mixed $4f^{13}$-$4f^{14}$ heterovalent complex or an intermediate $4f^{13.5}$ (2 x $Yb^{2.5+}$) complex. The isothermal magnetization at 2 K reaches a value of 2.2 Nμ_B, lower than the saturation value of M_{sat}= 4.54 $N\mu_B$ as expected from the presence of a magnetically anisotropic complex.[6] Variable-temperature alternating current (*ac*) susceptibility measurements were performed on the temperature range 2-10 K with a variable dc field up to 9 T to investigate the magnetic relaxation dynamics of **2**. However, no out-of-phase (χ'') signals were observed, indicating no slow relaxation of the magnetization.

4. Computational Details

To better understand the electronic structure of **2**, Density Functional Theory (DFT) calculations were undertaken. Three possible scenarios could exist: (i) electron localization on a single Yb center, resulting in localized Yb^{2+}/Yb^{3+} valency; (ii) reduction of the ligand resulting in three paramagnetic centers, $Yb^{3+}/ L^{\bullet-}/Yb^{3+}$; (iii) equal sharing of the electron by both Yb centers, i.e., $Yb^{2.5+}/Yb^{2.5+}$.

Scenario (i) is improbable as the X-ray crystal structure is centrosymmetric with no difference in the bonding at the Yb centers.

Scenario (ii): For the first set of calculations for **2**, a scenario was considered in which ligand reduction was caused by the additional electron, resulting in three paramagnetic centers: two equivalent Yb^{3+} centers with one radical ligand center (Figure SI 13). The following spin Hamiltonian was used to estimate two exchange coupling constants (J_1 and J_2) between the Yb and the radical and between the two Yb ions, respectively:

$$\hat{H} = - 2J_1 (\hat{S}_{Yb1}.\hat{S}_{rad}) - 2J_1 (\hat{S}_{Yb2}.\hat{S}_{rad}) - 2J_2 (\hat{S}_{Yb1}.\hat{S}_{Yb2})$$

For this, the anisotropic Yb^{3+} ions were replaced by the isotropic Gd^{3+} ions, calculating the energies of 4 different spin configurations (*vide infra*). To estimate the magnetic exchange value for Yb analogues ($Gd(f^7) \rightarrow Yb(f^1)$), a method known to reliably reproduce the experimental magnetic exchange value for lanthanide complexes was used where the estimated values were divided by 1/7 to obtain a qualitative value for the Yb analogues.[7, 8] The Gaussian16 suite of programs[9] with the hybrid B3LYP functional[10] and a triple-zeta quality basis set employing a Cundari-Stevens (CS) relativistic effective core potential on the Gd atom[11] and an SVP basis set for the rest of the atoms (C, N, and H) on the XRD structure was utilized.[12] The exchange coupling can be reliably estimated using Density Functional Theory (DFT) in combination with the broken symmetry approach.[13] A high spin solution (HS) with all spins up, two spin configurations with one of the lanthanide spins down (BS1-2), and one spin configuration with radical spin down (BS3) were included in the calculated spin configurations. All spin projections are given in Figure SI 13. A system of four equations with two unknowns was generated, which was then solved using linear equations followed by singular value decomposition.

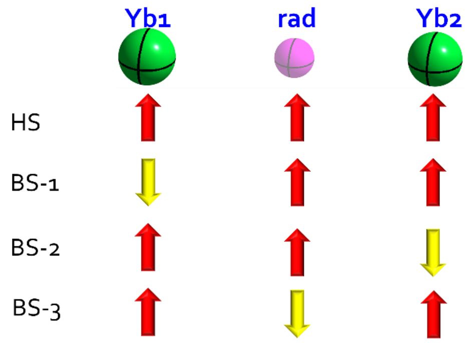

Figure SI 13. Spin configurations used to estimate the intramolecular magnetic exchange interactions for **2** in a scenario when the additional electron causes ligand reduction and, thus, radical center generation.

Ferromagnetic exchange between the Yb-Rad centers (J_1 = +6.0 cm^{-1}), and a very small antiferromagnetic exchange interaction between the two Yb ions (J_2 = -0.1 cm^{-1}) were estimated. This, relatively strong, ferromagnetic exchange interaction between lanthanide and radical center has been observed previously and attributed to strong charge transfer from the π* orbital of the ligand radical to empty metal orbitals (5*d*, 6*s* and 6*p*).[7] Magnetic measurements suggest that this is an unlikely scenario.

Scenario (iii): Thus, the last possibility, is where the additional electron is distributed equally between the two Yb centers. To explore the structural and bonding aspects for **2**, quantum theory of atoms in

molecules (QT-AIM)[14] and Wiberg bond index (WBI)[15] analysis using B3LYP/CSDZ(Yb)/SVP(N,C,H) were carried out.

In order to estimate the magnetic relaxation pathway and generate a magnetic blockade diagram (Figure SI 14), *ab initio* calculations using the MOLCAS 8.0 code were carried out. The calculations utilized basis sets [H.ANO-RCC...2s], [C.ANO-RCC...3s2p], [N.ANO-RCC...3s2p1d], and [Yb.ANO-RCC...7s6p4d2f1g] for the $^2F_{7/2}$ multiplet. Initially, guess orbitals were generated and then seven Yb^{3+}-based starting 4*f*-orbitals were selected for the CASSCF calculations. These calculations involved thirteen electrons in the seven active orbitals, generating an active space of CAS(13,7). Following this, 7 doublets were computed using the configuration interaction (CI) procedure within this active space. Subsequently, the RASSI-SO module was used to calculate the spin-orbit coupled states. After obtaining the spin-orbit states, the SINGLE_ANISO code was employed to extract the corresponding g-tensors for the eight low-lying Kramers Doublets. The Cholesky decomposition for two-electron integrals was utilized throughout the calculations to reduce disk space usage.[16]

To explore the possibility of the intervalence charge transfer (IVCT) and ligand-to-metal charge transfer (LMCT), time-dependent DFT (TDDFT) calculations were carried out on the optimized structure of complex **2** using the camB3LYP functional in ORCA 5.0.[17] The optimization has been performed by replacing Yb with Y, as the ionic radii for both are very close, using B3LYP/def2-SVP. The optimized structure is in good agreement with the XRD structure (Figure SI 15). The cyclohexyl groups were removed from the model complex to minimize the computational cost. The scalar relativistic Hamiltonian was included by using the zeroth-order regular approximation (ZORA) method. ZORA-contracted versions of basis sets SARC2-ZORA-QZVP were used for the Yb ions and the ZORA-def2-SVP basis set was used for the rest of the atoms.[18] In order to accurately calculate the 4*f* → 4*f* transitions and magnetic properties, state-averaged CASSCF (SA-CASSCF) calculations were conducted. Scalar relativistic effects were accounted for in these calculations using the second-order Douglas-Kroll-Hess (DKH) procedure.[19] To incorporate dynamic correlation, the converged wave functions underwent second-order N-electron valence perturbation theory (NEVPT2).[20] Spin-orbit coupling was considered using quasi-degenerate perturbation theory. The active space encompassed the 27 *f*-electrons in total sixteen orbitals (fourteen 4*f*-orbitals + two lowest energies 5*d* orbitals) averaged over all twelve spin-free states.

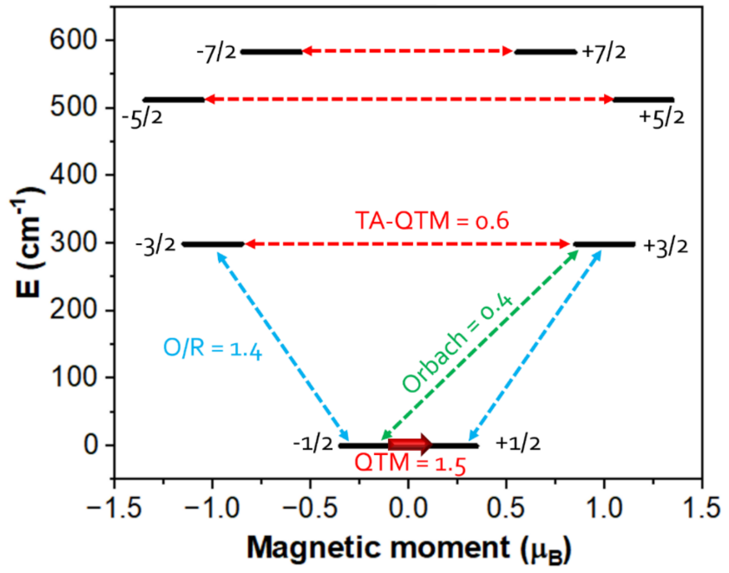

Figure SI 14. *Ab initio* computed magnetic blockade diagram illustrates magnetic blockade phenomena for **2**. The directional arrows within the diagram delineate connected energy states, with the numerical values denoting the matrix elements of the transverse moment. The acronyms QTM and TA-QTM correspond to quantum tunnelling of magnetization and thermally assisted QTM, respectively. Additionally, O/R denotes the

Orbach/Raman process. The numerical annotations above each arrow signify the transverse matrix elements for the transition magnetic moments. The ground state QTM value is estimated to be very large (for KD1; QTM = 1.5 and $g_{xx}/g_{yy}/g_{zz}$ = 5297/3.764/1.105) to cause relaxation via ground state and thus no slow relaxation of the magnetization as seen from experimental *ac* measurement.

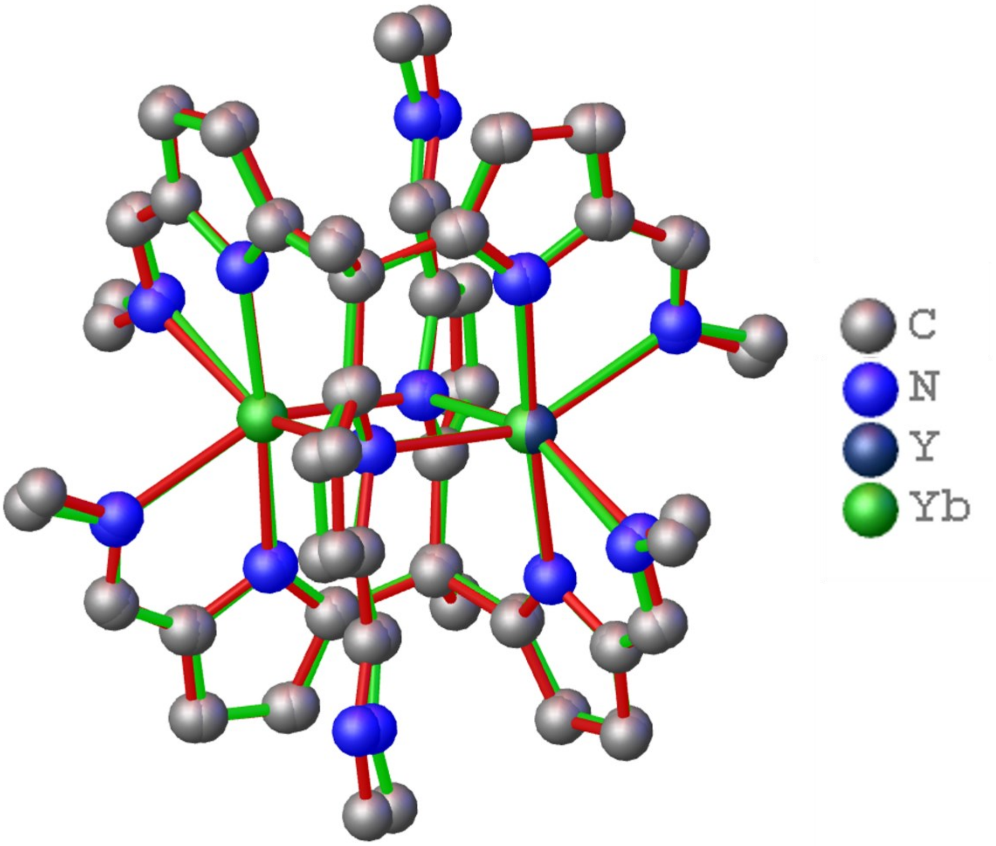

Figure SI 15. Overlap of DFT optimized and XRD structure for **2**. Yb was replaced by Y for the optimization.

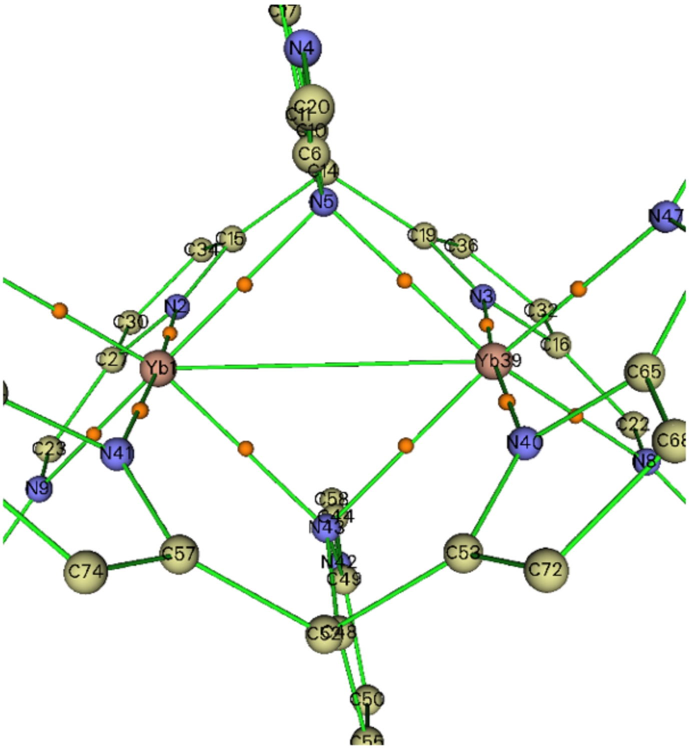

Figure SI 16. DFT-QTAIM computed contour map for **2**. Orange dots represent the bond center points (BCPs). The absence of BCP between both Yb ions suggests no bonding interaction between both Yb ions.

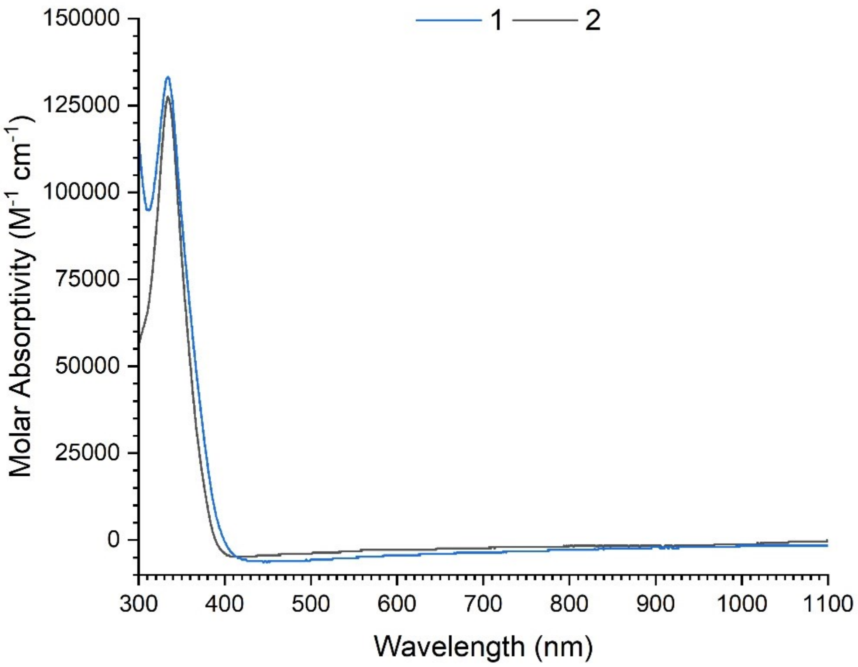

Figure SI 17. UV-vis-NIR spectra between 300 nm and 1100 nm of compounds **1** (BLUE) and **2** (BLACK) showing absorbances at 334 nm with molar absorptivities of 127600 M^{-1} cm^{-1} and 132800 M^{-1} cm^{-1} respectively. These are assigned a ligand based $\pi \rightarrow \pi^*$ transition.

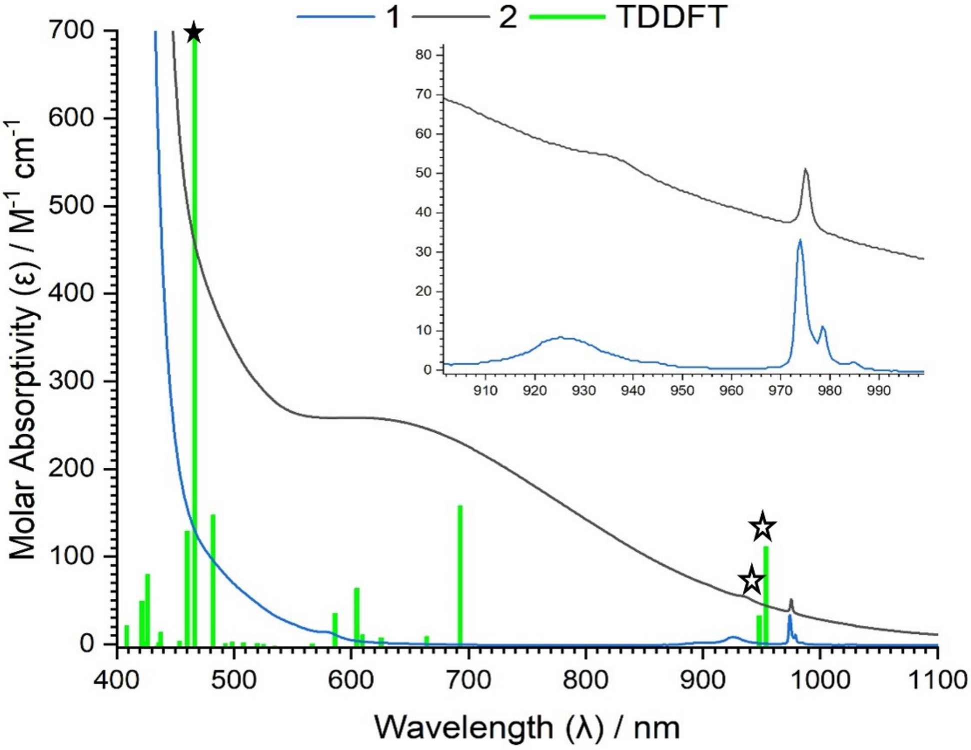

Figure SI 18. UV-Vis-NIR absorption spectrum of compounds **1** (Blue) and **2** (Black). Solutions were made in THF and scanned between 400 – 1100 nm. The results of TDDFT (TD-B3LYP) calculations of the $4f \rightarrow 4f$ transition energy of compound **2** is overlayed (Green bar). The peak at 466.9 nm with a black solid asterisk represents $4f \rightarrow 4f$ IVCT electronic transition. Meanwhile, the two peaks with the black hollow asterisks at 953.5 nm and 948.1 nm represent $4f \rightarrow 4f$ electronic transitions in the NIR region.

Note. Unlike TD-camB3LYP, TD-B3LYP reproduces the low energy transitions, indicating small contributions of 14.3% and 17.5% from $4f \rightarrow 4f$ orbitals for states 10 and 11 (at 953.5 nm and 948.1 nm, respectively. See Figure SI 18). One of the predicted transitions with large intensity at 466.9 nm with $4f \rightarrow 4f$ electronic transitions is seen, which is also seen for TD-camB3LYP but at lower energy (Figure 3 in the main manuscript).

State	Wavelength (nm) (*fosc*)	Donor	Acceptor	% Contribution
10	953.5 (0.0040)	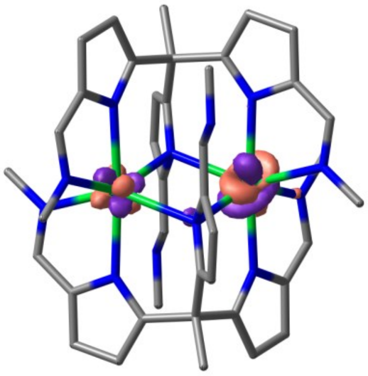	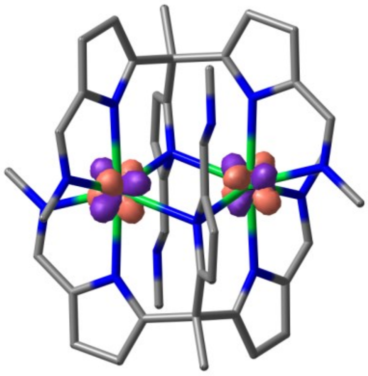	14.3
		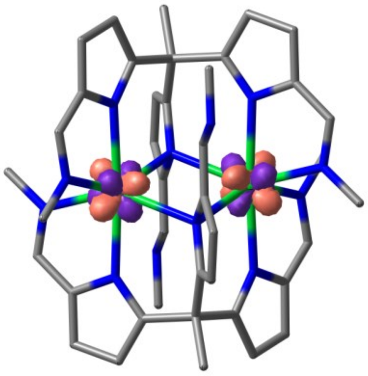	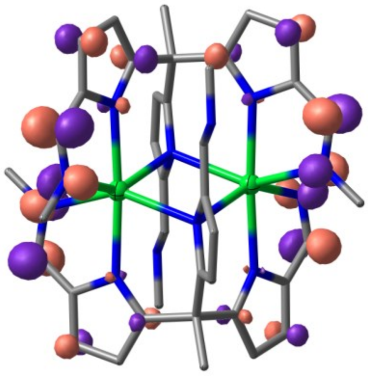	53.8
11	948.1 (0.0012)	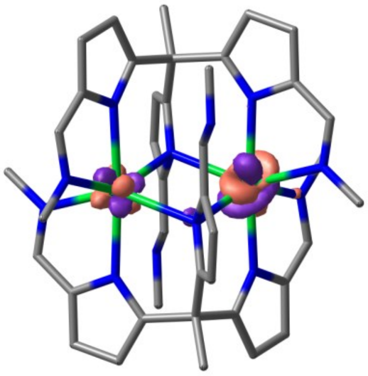	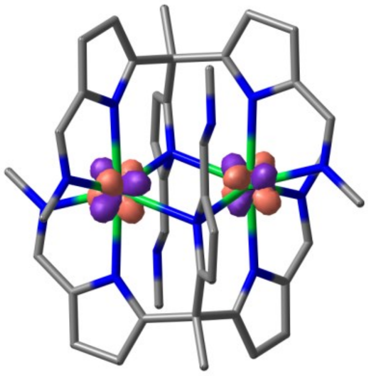	17.5
		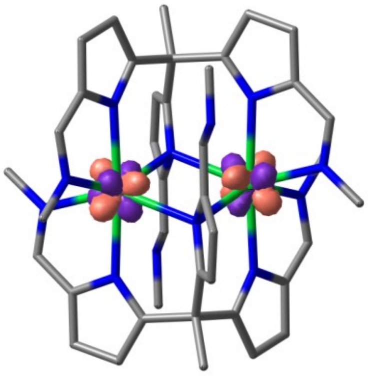	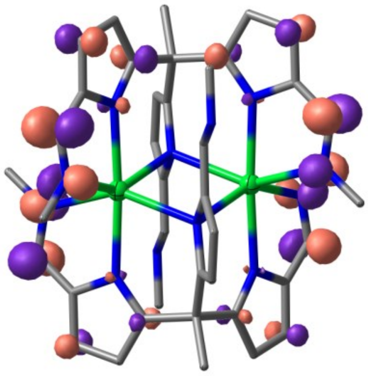	44.6

31	466.9 (0.0767)	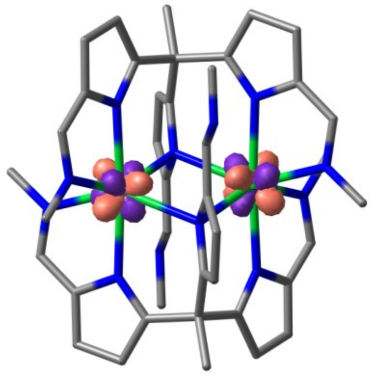	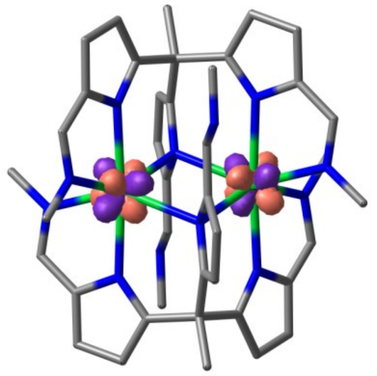	58.6

Table SI 3. Time-dependent DFT (TD-B3LYP) computed transition probabilities for complex **2** showing Yb(4*f*) → Yb(4*f*/5*d*) electronic transitions. For these states, any transitions with percentage contributions less than 10% are ignored and are not shown below.

5. Electrochemistry

To probe further the redox characteristics of **1**, cyclic voltammetry was carried out in THF using [$^{n}Bu_4N$][PF_6] electrolyte (Figure SI 18). The CV features a quasi-reversible reduction around $E_{1/2}$ = −2.49 V *vs* Fc^+/Fc. The quasi-reversibility of this redox event was confirmed by a Randles-Ševčik plot, which shows a deviation of the oxidation i_p from a linear fit when plotted against the square-root of the scan-rate (Figure SI 19). The shift in the peak-to-peak separation with scan-rate further supports the quasi-reversibility of this redox event rather than the deviation being due to surface-adsorbed species. This redox event has been assigned as the Yb^{3+}/Yb^{2+} metal reduction to a mixed-valent species, with the quasi-reversibility a result of the ligand rearrangement on reduction as seen in the X-ray crystal structure of **2**.

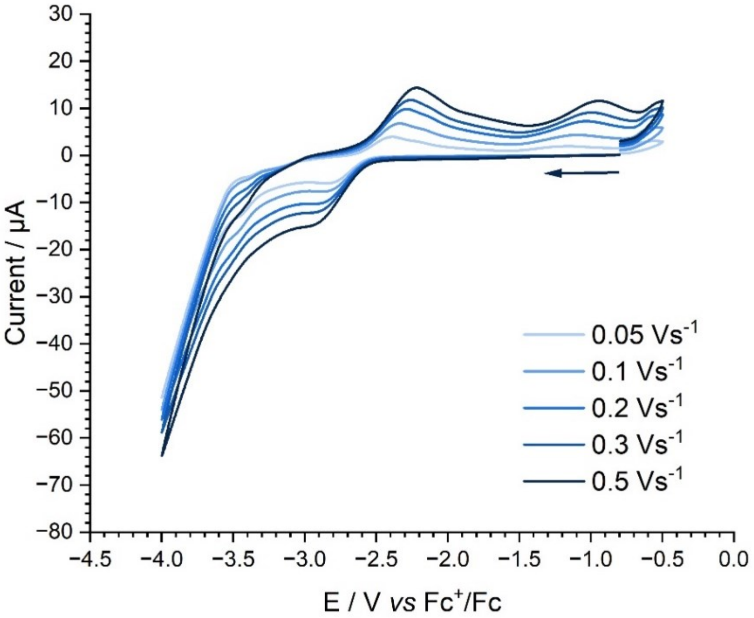

Figure SI 19. Cyclic voltammogram of complex **1**. A 5 mM solution of **1** in 0.1 M [$^{n}Bu_4N$][PF_6] electrolyte in THF using a platinum working electrode, platinum gauze counter electrode and silver pseudo-reference electrode. The spectra were referenced to ferrocene (Fc^+/Fc = 0 V) and run at scan-rates 0.05, 0.1, 0.2, 0.3 and 0.5 mV s^{-1}. The irreversible peak with E_{pc} = 3.37 V *vs* Fc^+/Fc (at 0.1 Vs^{-1}) becomes covered by the edge of the solvent window at higher scan-rates. The irreversible oxidation at E_{pa} = −1.10 V *vs* Fc^+/Fc (at 0.1 Vs^{-1}) is only present when scanning to potentials below 3.5 V *vs* Fc^+/Fc.

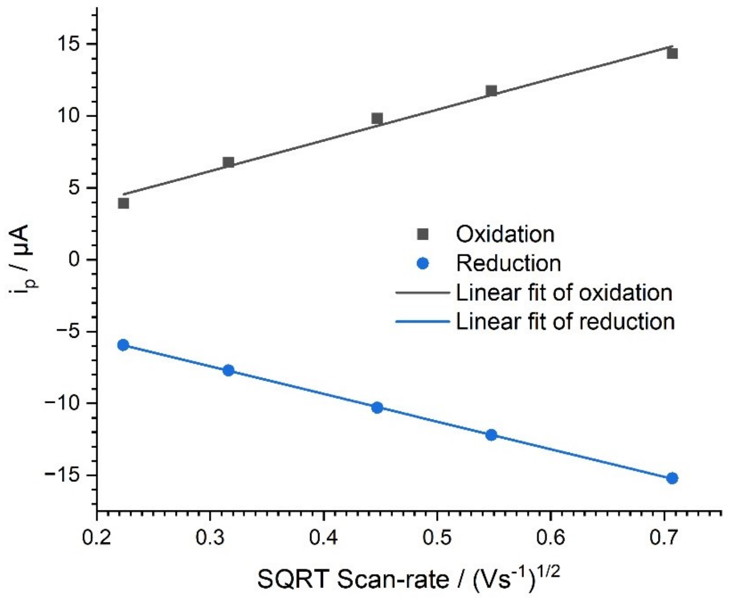

Figure SI 20. The Randles-Ševčik plot of the redox event at $E_{1/2}$ = −2.49 V *vs* Fc^+/Fc in compound **1**. The linear fit of the oxidation wave is shown in BLACK: adjusted r-square = 0.97919; residual sum of squares = 1.04501. The linear fit of the reduction wave is shown in BLUE: adjusted r-square = 0.99989; residual sum of squares = 0.00448.

The voltammogram also shows an irreversible reduction peak at E_{pc} = −3.37 V *vs* Fc^+/Fc. The identity of this event is either due to a second reduction to the monomeric Yb^{2+} species, analogous to complexes **4**, with the irreversibility a result of the monomerization of the dimeric complex, or it is a ligand reduction (Figure SI 20-21). The CV of H_3L displays a similar, irreversible reduction at E_{pc} = −3.38 V *vs* Fc^+/Fc that does not become reversible at increased scan-rates; however, this may be a result of H_2 generation from the acidic pyrrolyl-protons. The CV of **4** was measured and also shows only an irreversible reduction at E_{pc} = 3.30 V *vs* Fc^+/Fc. The absence of the acidic pyrrolyl-protons in **4** suggests this may be a ligand-based reduction analogous to that seen in **1**. There is also a small oxidation feature at E_{pa} = −1.79 V *vs* Fc^+/Fc that only occurs following the reduction wave at E_{pc} = −2.76 V *vs* Fc^+/Fc, suggesting decomposition of the reduction product on the electrode through an EC mechanism.

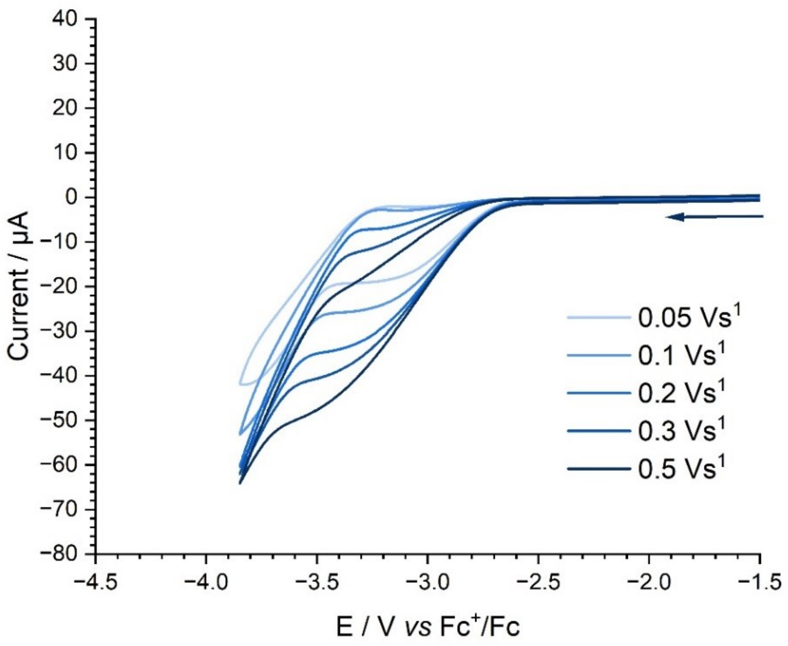

Figure SI 21. Cyclic voltammetry of a 10 mM solution of **H_3L** in 0.1 M [$^nBu_4N$][PF_6] electrolyte in THF using a platinum working electrode, platinum gauze counter electrode and silver pseudo-reference electrode. The spectra were referenced to ferrocene (Fc^+/Fc = 0 V) and run at scan-rates 0.05, 0.1, 0.2, 0.3 and 0.5 mV s^{-1}.

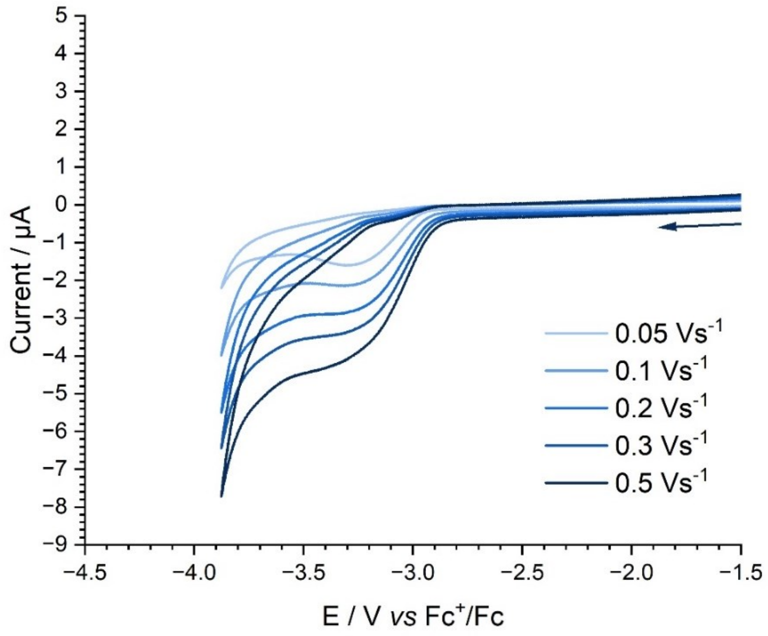

Figure SI 22. Cyclic voltammetry of a 10 mM solution of **4** in 0.1 M [$^nBu_4N$][PF_6] electrolyte in THF using a platinum working electrode, platinum gauze counter electrode and silver pseudo-reference electrode. The spectra were referenced to ferrocene (Fc^+/Fc = 0 V) and run at scan-rates 0.05, 0.1, 0.2, 0.3 and 0.5 mV s^{-1}.

6. Crystallographic details

	$[Yb(L)(py)]_2$, **1**
CCDC Number	2368421
Crystal data	
Chemical formula	$2(C_{82}H_{104}N_{14}Yb_2)$
M_r	3263.73
Crystal system, space group	Triclinic, $P\bar{1}$
Temperature (K)	100
a, *b*, *c* (Å)	15.2324 (6), 16.8025 (6), 19.2386 (7)
α, β, γ (°)	97.529 (2), 92.165 (2), 107.444 (2)
V (Å^3)	4641.5 (3)
Z	1
Radiation type	Mo *K*α
μ (mm^{-1})	2.05
Crystal size (mm)	0.11 × 0.08 × 0.04
Data collection	
Diffractometer	Bruker *APEX*-II CCD
Absorption correction	Multi-scan<br>*SADABS2016*/2 (Bruker,2016/2) was used for absorption correction. wR2(int) was 0.0579 before and 0.0510 after correction. The Ratio of minimum to maximum transmission is 0.9454. The l/2 correction factor is Not present.
T_{min}, T_{max}	0.706, 0.747
No. of measured, independent and observed [$I > 2\sigma(I)$] reflections	388684, 44734, 34616
R_{int}	0.050
$(\sin \theta/\lambda)_{max}$ (Å^{-1})	0.833
Refinement	
$R[F^2 > 2\sigma(F^2)]$, $wR(F^2)$, S	0.027, 0.061, 1.03
No. of reflections	44734
No. of parameters	885
H-atom treatment	H-atom parameters constrained
$\Delta\rangle_{max}$, $\Delta\rangle_{min}$ (e Å^{-3})	1.29, -0.67

	$[Yb(L)]_2[18c6(K)](py)_2$, **2**
CCDC Number	2368420
Crystal data	
Chemical formula	$C_{72}H_{94}N_{12}Yb_2 \cdot C_{12}H_{24}KO_6 \cdot 2(C_5H_5N)$
M_r	1935.28
Crystal system, space group	Triclinic, $P\bar{1}$
Temperature (K)	100
a, *b*, *c* (Å)	13.4582 (2), 14.4802 (2), 14.7107 (3)
α, β, γ (°)	118.756 (2), 97.504 (2), 109.685 (2)
V ($Å^3$)	2212.65 (8)
Z	1
Radiation type	Cu *K*α
μ (mm^{-1})	4.71
Crystal size (mm)	0.38 × 0.21 × 0.17
Data collection	
Diffractometer	SuperNova, Dual, Cu at home/near, Atlas
Absorption correction	Gaussian<br>*CrysAlis PRO* 1.171.42.49 (Rigaku Oxford Diffraction, 2022) Numerical absorption correction based on gaussian integration over a multifaceted crystal model Empirical absorption correction using spherical harmonics, implemented in SCALE3 ABSPACK scaling algorithm.
T_{min}, T_{max}	0.406, 0.907
No. of measured, independent and observed [$I > 2\sigma(I)$] reflections	47102, 9184, 8862
R_{int}	0.052
$(\sin \theta/\lambda)_{max}$ ($Å^{-1}$)	0.629
Refinement	
$R[F^2 > 2\sigma(F^2)]$, $wR(F^2)$, S	0.026, 0.068, 1.04
No. of reflections	9184
No. of parameters	554
H-atom treatment	H atoms treated by a mixture of independent and constrained refinement
$\Delta\rangle_{max}$, $\Delta\rangle_{min}$ (e $Å^{-3}$)	1.43, -1.10

	[Y(L)(py)], **4**
CCDC Number	2368423
Crystal data	
Chemical formula	$C_{41}H_{52}N_7Y{\cdot}C_3H_7$
M_r	774.89
Crystal system, space group	Monoclinic, $P2_1/n$
Temperature (K)	120
a, *b*, *c* (Å)	11.3318 (5), 28.1439 (11), 12.3575 (4)
β (°)	96.436 (4)
V (Å^3)	3916.2 (3)
Z	4
Radiation type	Mo *K*α
μ (mm^{-1})	1.53
Crystal size (mm)	0.67 × 0.16 × 0.12
Data collection	
Diffractometer	Xcalibur, Eos
Absorption correction	Analytical<br>*CrysAlis PRO* 1.171.42.49 (Rigaku Oxford Diffraction, 2022) Analytical numeric absorption correction using a multifaceted crystal model based on expressions derived by R.C. Clark & J.S. Reid. (Clark, R. C. & Reid, J. S. (1995). Acta Cryst. A51, 887-897) Empirical absorption correction using spherical harmonics, implemented in SCALE3 ABSPACK scaling algorithm.
T_{min}, T_{max}	0.863, 0.960
No. of measured, independent and observed [$I > 2\sigma(I)$] reflections	38761, 7673, 5789
R_{int}	0.063
$(\sin \theta/\lambda)_{max}$ (Å^{-1})	0.617
Refinement	
$R[F^2 > 2\sigma(F^2)]$, $wR(F^2)$, S	0.052, 0.102, 1.08
No. of reflections	7673
No. of parameters	688
No. of restraints	53
H-atom treatment	H atoms treated by a mixture of independent and constrained refinement
$\Delta\rangle_{max}$, $\Delta\rangle_{min}$ (e Å^{-3})	1.07, -0.47

	$[Yb(L)]_2[crypt(K)]$, **5**
CCDC Number	2374047
Crystal data	
Chemical formula	$C_{36}H_{47}N_6Yb{\cdot}C_9H_{18}K_{0.5}NO_3$
M_r	944.63
Crystal system, space group	Monoclinic, $C2/c$
Temperature (K)	120
a, b, c (Å)	15.3748 (6), 22.0364 (14), 27.1363 (9)
β (°)	104.784 (4)
V (Å^3)	8889.6 (7)
Z	8
Radiation type	Cu $K\alpha$
μ (mm^{-1})	4.67
Crystal size (mm)	0.23 × 0.05 × 0.03
Data collection	
Diffractometer	SuperNova, Dual, Cu at home/near, Atlas
Absorption correction	Gaussian<br>*CrysAlis PRO* 1.171.42.49 (Rigaku Oxford Diffraction, 2022) Numerical absorption correction based on gaussian integration over a multifaceted crystal model Empirical absorption correction using spherical harmonics, implemented in SCALE3 ABSPACK scaling algorithm.
T_{min}, T_{max}	0.315, 1.000
No. of measured, independent and observed [$I > 2\sigma(I)$] reflections	45603, 8438, 5723
R_{int}	0.104
$(\sin \theta/\lambda)_{max}$ (Å^{-1})	0.610
Refinement	
$R[F^2 > 2\sigma(F^2)]$, $wR(F^2)$, S	0.052, 0.114, 1.01
No. of reflections	8438
No. of parameters	603
No. of restraints	560
H-atom treatment	H-atom parameters constrained
	$w = 1/[\sigma^2(F_o^2) + (0.0365P)^2 + 32.2028P]$<br>where $P = (F_o^2 + 2F_c^2)/3$
$\Delta\rangle_{max}$, $\Delta\rangle_{min}$ (e Å^{-3})	0.61, -1.06

7. DFT optimized xyz coordinates for complex 2 (without K-18-Crown-6)

Yb	0.001449000	1.810328000	-0.000566000
Yb	-0.001448000	-1.810305000	0.000566000
N	-2.333468000	1.899741000	-0.345058000
N	2.951709000	-0.002069000	-3.348284000
N	-0.382933000	0.000197000	-1.738108000
N	-2.336415000	-1.896253000	-0.344732000
N	-0.887719000	-3.511946000	1.405612000
N	-0.882095000	3.513245000	1.404646000
C	2.071478000	-0.001602000	-2.418713000
H	2.371354000	-0.001761000	-1.356919000
C	-1.585926000	0.000905000	-2.462825000
C	-3.011967000	0.002078000	-1.889783000
C	0.093836000	-0.001045000	-3.987108000
H	0.676515000	-0.001801000	-4.905598000
C	0.642861000	-0.000948000	-2.713407000
C	-1.303796000	0.000167000	-3.830054000
H	-2.031681000	0.000454000	-4.636047000
C	-2.178960000	3.714700000	1.254538000
H	-2.684694000	4.507853000	1.829011000
C	-3.301457000	-1.243568000	-1.038386000
C	4.342924000	-0.002811000	-2.968119000
H	4.520019000	-0.002817000	-1.874691000
C	-3.299602000	1.247862000	-1.037963000
C	-4.561338000	-1.837268000	-0.782704000
H	-5.520256000	-1.525968000	-1.191797000
C	-2.958598000	-2.909134000	0.377162000
C	-2.954260000	2.913052000	0.377453000
C	-4.017578000	0.003103000	-3.066419000
H	-3.893176000	-0.893978000	-3.689322000
H	-5.048109000	0.004129000	-2.691733000
C	-2.184709000	-3.711820000	1.254556000
C	-0.151552000	-4.326933000	2.351220000
H	-0.790785000	-5.095804000	2.826062000
C	-0.144330000	4.326911000	2.350154000
H	-0.781821000	5.097733000	2.824177000
C	-4.343801000	-2.895910000	0.110798000
H	-5.089165000	-3.574372000	0.526719000
C	-4.558794000	1.842542000	-0.781195000
H	-5.518311000	1.532047000	-1.189496000
C	-4.339688000	2.900946000	0.112207000
H	-5.084190000	3.579920000	0.528833000
H	-2.691773000	-4.504539000	1.828476000
N	2.333473000	-1.899707000	0.345013000
N	-2.951713000	0.001856000	3.348276000
N	0.382932000	-0.000179000	1.738108000
N	2.336412000	1.896285000	0.344779000
N	0.887736000	3.511985000	-1.405577000
N	0.882080000	-3.513206000	-1.404679000
C	-2.071480000	0.001554000	2.418708000
H	-2.371353000	0.001783000	1.356912000

C	1.585925000	-0.000948000	2.462827000
C	3.011966000	-0.002081000	1.889785000
C	-0.093841000	0.000893000	3.987107000
H	-0.676521000	0.001583000	4.905596000
C	-0.642864000	0.000884000	2.713405000
C	1.303792000	-0.000288000	3.830056000
H	2.031674000	-0.000614000	4.636050000
C	2.178953000	-3.714636000	-1.254614000
H	2.684687000	-4.507767000	-1.829119000
C	3.301448000	1.243600000	1.038441000
C	-4.342927000	0.002540000	2.968106000
H	-4.520019000	0.002677000	1.874678000
C	3.299611000	-1.247829000	1.037912000
C	4.561325000	1.837326000	0.782806000
H	5.520237000	1.526031000	1.191915000
C	2.958596000	2.909194000	-0.377075000
C	2.954263000	-2.912991000	-0.377537000
C	4.017581000	-0.003139000	3.066419000
H	3.893030000	0.893815000	3.689475000
H	5.048111000	-0.003931000	2.691730000
C	2.184718000	3.711884000	-1.254476000
C	0.151579000	4.326990000	-2.351176000
H	0.790819000	5.095860000	-2.826009000
C	0.144307000	-4.326856000	-2.350195000
H	0.781790000	-5.097683000	-2.824222000
C	4.343791000	2.895998000	-0.110663000
H	5.089152000	3.574487000	-0.526541000
C	4.558809000	-1.842475000	0.781088000
H	5.518332000	-1.531969000	1.189367000
C	4.339699000	-2.900861000	-0.112332000
H	5.084203000	-3.579809000	-0.528997000
H	2.691783000	4.504627000	-1.828361000
H	-3.891349000	0.899985000	-3.689245000
H	4.849231000	0.880714000	-3.399792000
H	4.848260000	-0.886982000	-3.399612000
H	3.891505000	-0.900147000	3.689092000
H	-0.284640000	-3.702323000	-3.153733000
H	-0.700974000	-4.838930000	-1.856465000
H	0.279425000	-3.703008000	3.154126000
H	0.692157000	-4.841523000	1.857386000
H	-4.848331000	0.886617000	3.399714000
H	-4.849170000	-0.881079000	3.399662000
H	0.700945000	4.838993000	1.856420000
H	0.284628000	3.702390000	3.153696000
H	-0.692127000	4.841582000	-1.857339000
H	-0.279403000	3.703079000	-3.154091000

8. **Input for TD-camB3LYP calculation.**

```
! CAM-B3LYP ZORA ZORA-def2-SVP AUTOAUX RIJCOSX veryslowconv notrah LARGEPRINT

%tddft
nroots 100
maxdim 20
end
%scf
maxiter 1500
DIISMaxEq 40
directresetfreq 1
DampFac 0.98
DampErr 0.05
end

%basis
newgto Yb "sarc2-zora-QZVP" end
newauxgto Yb "autoaux" end
end

* xyz -1 2
XYZ coordinate for complex 2
*
```

9. **Input for CASSCF calculation**:

```
! DKH DKH-def2-SVP AUTOAUX LARGEPRINT
!Moread
!NoIter
%MoInp "/path/TD-camb3lyp.gbw"

%basis
newgto Yb "sarc2-DKH-QZVP" end
newauxcgto Yb "autoaux" end
end

%rel
picturechange 2
end

%casscf
nel 27
norb 16 (Note: This includes 14 4f and 2 lowest energies 5d orbitals)
mult 2
nroots 12
trafostep rimo
printwf true
nevpt2 true
nevpt
D4Tpre 1e-13
```

```
end
rel
dosoc true
domagnetization true
dosusceptibility true
SUSStatFieldMIN 0.0
SUSStatFieldMAX 10000.0
SUSStatFieldNPoints 2
gtensor true
printlevel 3
end
end

%SCF
  MAXITER 3000
   MAXCORE 4500
  MaxIntMem 30000
end

%scf rotate (command to swap all f and 2 lowest energies 5d orbitals into the active CAS orbitals)end
end

* xyz -1 2
```
XYZ coordinate for complex **2**

```
*

%tddft
 Nroots=100
 Maxdim=20
 end
```

10. References

(1) Fox, O. D.; Rolls, T. D.; Drew, M. G. B.; Beer, P. D. The binding of difunctional neutral guest molecules by novel bis(tripyrrolyl) cryptands. *Chem. Commun.* **2001**, (17), 1632-1633. Boyle, T. J.; Yonemoto, D. T.; Sears, J. M.; Treadwell, L. J.; Bell, N. S.; Cramer, R. E.; Neville, M. L.; Stillman, G. A. K.; Bingham, S. P. Synthesis, characterization, and utility of trifluoroacetic acid lanthanide precursors for production of varied phase fluorinated lanthanide nanomaterials. *Polyhedron* **2017**, *131*, 59-73.

(2) Sheldrick, G. Crystal structure refinement with SHELXL. *Acta Crystallogr. Sect. C* **2015**, *71* (1), 3-8. Sheldrick, G. SHELXT - Integrated space-group and crystal-structure determination. *Acta Crystallogr. Sect. A* **2015**, *71* (1), 3-8.

(3) Dolomanov, O. V.; Bourhis, L. J.; Gildea, R. J.; Howard, J. A. K.; Puschmann, H. OLEX2: a complete structure solution, refinement and analysis program. *J. Appl. Crystallogr.* **2009**, *42* (2), 339-341.

(4) Macrae, C. F.; Sovago, I.; Cottrell, S. J.; Galek, P. T. A.; McCabe, P.; Pidcock, E.; Platings, M.; Shields, G. P.; Stevens, J. S.; Towler, M.; et al. Mercury 4.0: from visualization to analysis, design and prediction. *J. Appl. Crystallogr.* **2020**, *53* (1), 226-235.

(5) Hart, J. S.; Nichol, G. S.; Love, J. B. Directed secondary interactions in transition metal complexes of tripodal pyrrole imine and amide ligands. *Dalton Trans.* **2012**, *41* (19), 5785-5788.

(6) Regincós Martí, E.; Canaj, A. B.; Sharma, T.; Celmina, A.; Wilson, C.; Rajaraman, G.; Murrie, M. Importance of an Axial Ln^{III}–F Bond across the Lanthanide Series and Single-Molecule Magnet Behavior in the Ce and Nd Analogues. *Inorg. Chem.* **2022**, *61* (26), 9906-9917.

(7) Singh, M. K.; Yadav, N.; Rajaraman, G. Record high magnetic exchange and magnetization blockade in $Ln_2@C_{79}N$ (Ln = Gd(III) and Dy(III)) molecules: a theoretical perspective. *Chem. Commun.* **2015**, *51* (100), 17732-17735.

(8) Singh, M. K.; Rajaraman, G. Acquiring a record barrier height for magnetization reversal in lanthanide encapsulated fullerene molecules using DFT and ab initio calculations. *Chem. Commun.* **2016**, *52* (97), 14047-14050.

(9) *Gaussian 16 Rev. C.01*; Wallingford, CT, 2016.

(10) Becke, A. D. Density-functional thermochemistry. III. The role of exact exchange. *J. Chem. Phys.* **1993**, *98* (7), 5648-5652. Lee, C.; Yang, W.; Parr, R. G. Development of the Colle-Salvetti correlation-energy formula into a functional of the electron density. *Phys. Rev. B* **1988**, *37* (2), 785-789. Stephens, P. J.; Devlin, F. J.; Chabalowski, C. F.; Frisch, M. J. Ab Initio Calculation of Vibrational Absorption and Circular Dichroism Spectra Using Density Functional Force Fields. *J. Phys. Chem.* **1994**, *98* (45), 11623-11627.

(11) Cundari, T. R.; Stevens, W. J. Effective core potential methods for the lanthanides. *J. Chem. Phys.* **1993**, *98* (7), 5555-5565.

(12) Schäfer, A.; Horn, H.; Ahlrichs, R. Fully optimized contracted Gaussian basis sets for atoms Li to Kr. *J. Chem. Phys.* **1992**, *97* (4), 2571-2577.

(13) Hazra, S.; Bhattacharya, S.; Singh, M. K.; Carrella, L.; Rentschler, E.; Weyhermueller, T.; Rajaraman, G.; Mohanta, S. Syntheses, Structures, Magnetic Properties, and Density Functional Theory Magneto-Structural Correlations of Bis(μ-phenoxo) and Bis(μ-phenoxo)-μ-acetate/Bis(μ-phenoxo)-bis(μ-acetate) Dinuclear $Fe^{III}Ni^{II}$ Compounds. *Inorg. Chem.* **2013**, *52* (22), 12881-12892. Upadhyay, A.; Rajpurohit, J.; Kumar-Singh, M.; Dubey, R.; Kumar Srivastava, A.; Kumar, A.; Rajaraman, G.; Shanmugam, M. Hydroxo-Bridged Dimers of Oxo-Centered Ruthenium(III) Triangle: Synthesis and Spectroscopic and Theoretical Investigations. *Chem. Eur. J.* **2014**, *20*, 6061-6070. McDonald, C.; Sanz, S.; Brechin, E. K.; Singh, M. K.; Rajaraman, G.; Gaynor, D.; Jones, L. F. High nuclearity Ni(II) cages from hydroxamate ligands. *RSC Adv.* **2014**, *4* (72), 38182-38191. Caballero-Jiménez, J.; Habib, F.; Ramírez-Rosales, D.; Grande-Aztatzi, R.; Merino, G.; Korobkov, I.; Singh, M. K.; Rajaraman, G.; Reyes-Ortega, Y.; Murugesu, M. Inducing magnetic communication in caged dinuclear Co(II) systems. *Dalton Trans.* **2015**, *44* (18), 8649-8659. Singh, M. K.; Rajeshkumar, T.; Kumar, R.; Singh, S. K.; Rajaraman, G. Role of (1,3) {Cu-Cu} Interaction on the Magneto-Caloric Effect of Trinuclear {Cu^{II}-Gd^{III}-Cu^{II}} Complexes: Combined DFT and Experimental Studies. *Inorg. Chem.* **2018**, *57* (4), 1846-1858. Ghosh, S.; Mandal, S.; Singh, M. K.; Liu, C.-M.; Rajaraman, G.; Mohanta, S. Experimental and theoretical exploration of

magnetic exchange interactions and single-molecule magnetic behaviour of bis(η^1:η^2:μ_2-carboxylate)Gd^{III}_2/Dy^{III}_2 systems. *Dalton Trans.* **2018**, *47* (33), 11455-11469. Peng, Y.; Singh, M. K.; Mereacre, V.; Anson, C. E.; Rajaraman, G.; Powell, A. K. Mechanism of magnetisation relaxation in {$M^{III}_2Dy^{III}_2$} (M = Cr, Mn, Fe, Al) "Butterfly" complexes: how important are the transition metal ions here? *Chem. Sci.* **2019**, *10* (21). Singh, M. K.; Rajaraman, G. Can CH···π Interactions Be Used To Design Single-Chain Magnets? *Chem. Eur. J.* **2015**, *21*, 980-983.
(14) Bader, R. F. W. *Atoms in Molecules - A Quantum Theory*; 1990.
(15) Wiberg, K. B. Application of the pople-santry-segal CNDO method to the cyclopropylcarbinyl and cyclobutyl cation and to bicyclobutane. *Tetrahedron* **1968**, *24* (3), 1083-1096. Mayer, I. Bond order and valence indices: A personal account. *J. Comput. Chem.* **2007**, *28* (1), 204-221. Harper, L. K.; Shoaf, A. L.; Bayse, C. A. Predicting Trigger Bonds in Explosive Materials through Wiberg Bond Index Analysis. *ChemPhysChem* **2015**, *16* (18), 3886-3892.
(16) Aquilante, F.; Autschbach, J.; Carlson, R. K.; Chibotaru, L. F.; Delcey, M. G.; De Vico, L.; Fdez. Galván, I.; Ferré, N.; Frutos, L. M.; Gagliardi, L.; et al. Molcas 8: New capabilities for multiconfigurational quantum chemical calculations across the periodic table. *J. Comput. Chem.* **2016**, *37* (5), 506-541. Duncan, J. A. MOLCAS 7.2. *J. Am. Chem. Soc.* **2009**, *131* (6), 2416-2416. Veryazov, V.; Widmark, P.-O.; Serrano-Andrés, L.; Lindh, R.; Roos, B. O. 2MOLCAS as a development platform for quantum chemistry software. *Int. J. Quantum Chem* **2004**, *100* (4), 626-635.. Karlström, G.; Lindh, R.; Malmqvist, P.-Å.; Roos, B. O.; Ryde, U.; Veryazov, V.; Widmark, P.-O.; Cossi, M.; Schimmelpfennig, B.; Neogrady, P.; et al. MOLCAS: a program package for computational chemistry. *Com. Mat. Sci.* **2003**, *28* (2), 222-239. Aquilante, F.; De Vico, L.; Ferré, N.; Ghigo, G.; Malmqvist, P.-å.; Neogrády, P.; Pedersen, T. B.; Pitoňák, M.; Reiher, M.; Roos, B. O.; et al. MOLCAS 7: The Next Generation. *J. Comput. Chem.* **2010**, *31* (1), 224-247.
(17) Roy, M. D.; Gompa, T. P.; Greer, S. M.; Jiang, N.; Nassar, L. S.; Steiner, A.; Bacsa, J.; Stein, B. W.; La Pierre, H. S. Intervalence Charge Transfer in Nonbonding, Mixed-Valence, Homobimetallic Ytterbium Complexes. *J. Am. Chem. Soc.* **2024**, *146* (8), 5560-5568. Neese, F. Software update: The ORCA program system—Version 5.0. *WIREs Comput. Mol. Sci.* **2022**, *12* (5), e1606.
(18) van Wüllen, C. Molecular density functional calculations in the regular relativistic approximation: Method, application to coinage metal diatomics, hydrides, fluorides and chlorides, and comparison with first-order relativistic calculations. *J. Chem. Phys.* **1998**, *109* (2), 392-399.
(19) Heß, B. A.; Marian, C. M.; Wahlgren, U.; Gropen, O. A mean-field spin-orbit method applicable to correlated wavefunctions. *Chem. Phys. Lett.* **1996**, *251* (5), 365-371.
(20) Angeli, C.; Cimiraglia, R.; Evangelisti, S.; Leininger, T.; Malrieu, J.-P. Introduction of n-electron valence states for multireference perturbation theory. *J. Chem. Phys.* **2001**, *114* (23), 10252-10264.
